# Identifying Driver Genomic Alterations in Cancers by Searching Minimum-Weight, Mutually Exclusive Sets

**DOI:** 10.1371/journal.pcbi.1004257

**Published:** 2015-08-28

**Authors:** Songjian Lu, Kevin N. Lu, Shi-Yuan Cheng, Bo Hu, Xiaojun Ma, Nicholas Nystrom, Xinghua Lu

**Affiliations:** 1 Department of Biomedical Informatics, University of Pittsburgh, Pittsburgh, Pennsylvania, United States of America; 2 Department of Neurology, Northwestern Brain Tumor Institute, Center for Genetic Medicine, The Robert H. Lurie Comprehensive Cancer Center, Northwestern University Feinberg School of Medicine, Chicago, Illinois, United States of America; 3 Pittsburgh Supercomputing Center, Pittsburgh, Pennsylvania, United States of America; ETH Zurich, SWITZERLAND

## Abstract

An important goal of cancer genomic research is to identify the driving pathways underlying disease mechanisms and the heterogeneity of cancers. It is well known that somatic genome alterations (SGAs) affecting the genes that encode the proteins within a common signaling pathway exhibit mutual exclusivity, in which these SGAs usually do not co-occur in a tumor. With some success, this characteristic has been utilized as an objective function to guide the search for driver mutations within a pathway. However, mutual exclusivity alone is not sufficient to indicate that genes affected by such SGAs are in common pathways. Here, we propose a novel, signal-oriented framework for identifying driver SGAs. First, we identify the perturbed cellular signals by mining the gene expression data. Next, we search for a set of SGA events that carries strong information with respect to such perturbed signals while exhibiting mutual exclusivity. Finally, we design and implement an efficient exact algorithm to solve an NP-hard problem encountered in our approach. We apply this framework to the ovarian and glioblastoma tumor data available at the TCGA database, and perform systematic evaluations. Our results indicate that the signal-oriented approach enhances the ability to find informative sets of driver SGAs that likely constitute signaling pathways.

## Author Summary

An important goal of studying cancer genomics is to identify critical pathways that, when perturbed by somatic genomic alterations (SGAs) such as somatic mutations, copy number alterations and epigenomic alterations, cause cancers and underlie different clinical phenotypes. In this study, we present a framework for discovering perturbed signaling pathways in cancers by integrating genome alteration data and transcriptomic data from the Cancer Genome Atlas (TCGA) project. Since gene expression in a cell is regulated by cellular signaling systems, we used transcriptomic changes to reveal perturbed cellular signals in each tumor. We then combined the genomic alteration data to search for SGA events across multiple tumors that affected a common signal, thus identifying the candidate members of cancer pathways. Our results demonstrate the advantage of the signal-oriented pathway approach over previous methods.

## Introduction

Somatic genome alterations (SGAs) such as somatic mutations, somatic copy number alterations and epigenomic alterations are major causes of cancers[1–3]. In general, SGAs in a tumor can be divided into two types: those that affect cellular signaling proteins, perturb the cellular signaling system, and eventually contribute to cancer initiation and progression are called *driver SGAs*; and those that do not directly contribute to cancer development are designated as *passenger SGAs*. A fundamental problem of cancer-genome research is to identify signaling pathways that, when perturbed by driver SGAs, lead to cancer development or affect clinical outcomes for patients. Identification of such pathways will not only advance our understanding of the disease mechanisms underlying cancer, but will also provide guidance for the precision treatment of cancer patients.

**Fig 1:**
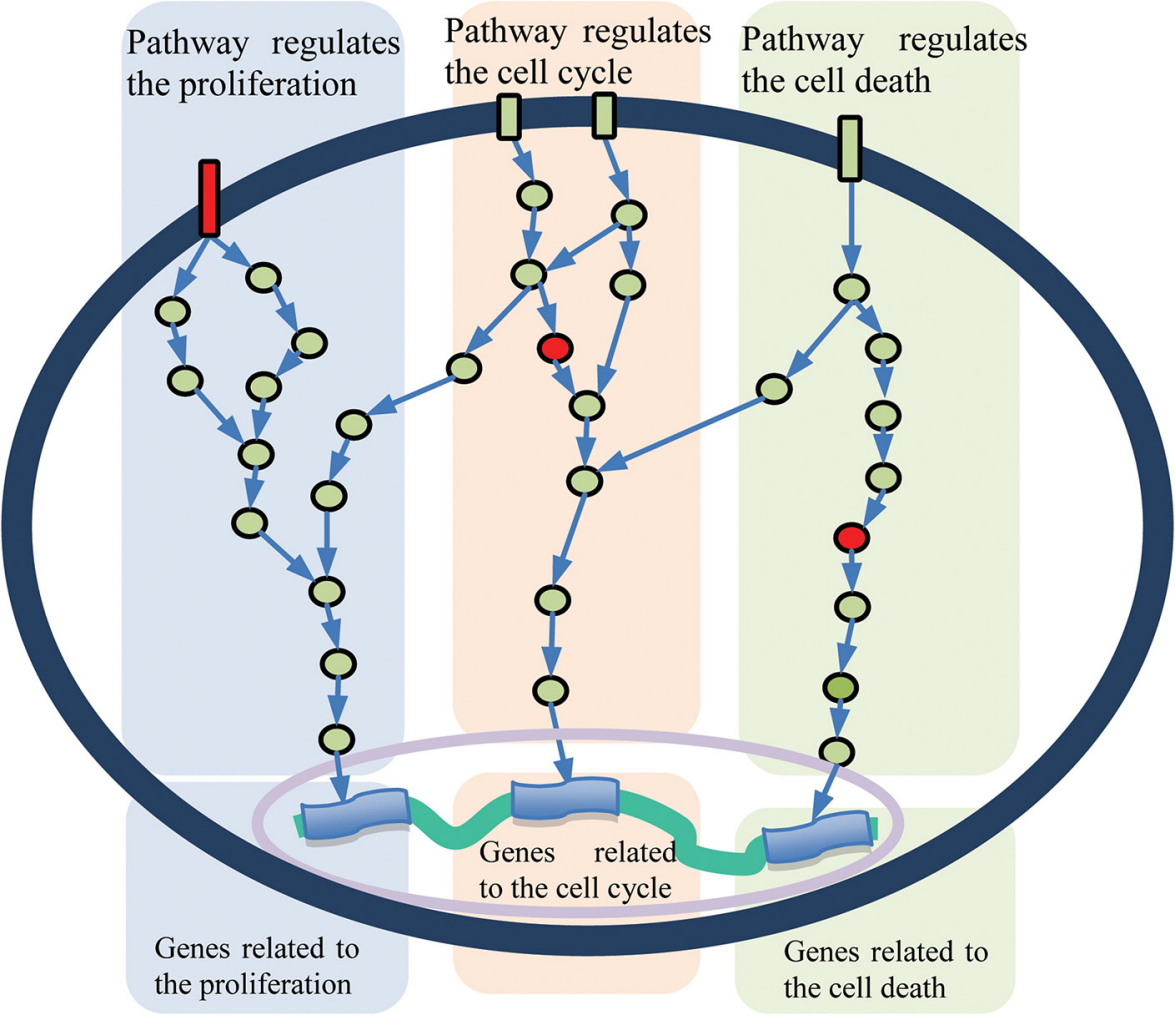
A diagram reflecting the signaling systems of a cancer cell. A signal pathway usually consists of a cascade of proteins, represented as rectangle (receptors) and circle (intracellular) nodes. Different pathways transmit signals regulating expression of distinct sets of target genes. In cancers, a pathway can be perturbed by an SGA event affecting a member protein (indicated by a red node); a cancer cell usually has multiple pathways perturbed.

In a cell, signaling pathways detect and transmit cellular signals to maintain cellular homeostasis; often, such signals eventually regulate the transcription of genes in order to initiate certain biological processes. For example, the signal transmitted by a growth factor usually leads to the transcription of genes involved in cell proliferation (Fig 1). As such, the impact of an SGA affecting a signaling protein in a tumor often manifests as an expression signature embedded in the expression profile of the tumor. For example, a mutation leading to constitutive activation of the epidermal growth factor receptor (*EGFR*) gene may lead to over-expression of its target genes. Thus, the gene expression profile of a cell at a given time reflects the state of its cellular signaling system, although it is a convoluted response to all active signals. Inferring the state of an individual pathway requires the deconvolution of the signals embedded in its gene expression data. The Cancer Genome Atlas (TCGA) has collected the most comprehensive genome-scale data to date, including somatic mutations, copy number variations, and gene expression from a large number of different types of cancers. By simultaneously capturing SGAs and gene expression data from each tumor, the TCGA data reflect the *cause* and *readout* of perturbed signaling pathways, thus providing a unique opportunity for studying cancer pathways.

Identification of perturbed and critical signaling pathways using the TCGA data is challenging in the following ways. First, as a cancer cell usually hosts dozens to hundreds of SGAs, we need to identify the small number of driver SGAs among the large number of passenger SGAs within a given tumor. Current approaches for identifying driver SGAs mainly concentrate on those that occur beyond random chance in a patient population[4–7]. These approaches would fail to find low-prevalence SGAs that affect a specific cancer-driving pathway. The second challenge is caused by the heterogeneity of mutations in tumor cells, in that few tumors have identical SGA patterns. One reason for this phenomenon is that a signal pathway can be perturbed in multiple locations[8], and different tumors may share a common aberrant pathway but exhibit SGAs affecting distinct proteins on the pathway. Thus, it is a challenge to determine if distinct SGA events in different tumors affect a common pathway. Finally, a cancer results from perturbations in multiple pathways[2], and distinct combinations of pathway perturbation underlie the heterogeneity of cancers[2] in terms of clinical phenotype. Thus, it is a challenge to determine, among multiple SGAs and multiple aberrant pathways in a tumor, which SGA affects which pathway.

Researchers have developed various approaches to search for driving pathways using TCGA by exploiting different properties of tumor cells[9–11], including mutual exclusivity[12–14], which is the observed phenomenon that SGA events affecting the proteins within a signaling pathway seldom co-occur in a tumor. A natural explanation for this phenomenon is that, if one mutation is sufficient to perturb the signal of a pathway and leads to the development of cancer, perturbation of other proteins is not required, and therefore co-occurrence of perturbations is seldom observed. This property is observed in different types of cancer cells and pathways[4,13,15,16].

While it is the case that SGAs affecting the genes within a common pathway tend to be mutually exclusive, the reverse may not necessarily be the case, that is, finding a set of mutually exclusive SGAs does not ensures that their corresponding proteins are in the same pathway. Current mutual-exclusivity-based methods[12–14,16,17] concentrate on finding a set of SGAs of size *k*, such that the set covers as many tumors as possible while minimizing overlapping cover (thus maximizing mutual exclusivity). As the numbers of tumors and SGAs examined by contemporary genome technology increase, it becomes increasingly easier to find a set of unrelated SGAs that covers a certain number of tumors while exhibiting mutually exclusivity due to the heterogeneity of tumors. To address this shortcoming, Zhao et al[14] further considered the co-expression of SGA-affected genes in order to enhance the search of pathways. The intuition underlying their approach is that proteins within a signaling pathway tend to be co-expressed, so the correlation of the expressions of the candidate genes of a pathway can be used as another objective function to guide pathway search. Both mutual exclusivity and co-expression of SGAs are auxiliary properties of a signaling pathway, but they are not sufficient to indicate whether the SGAs affect a common cellular signal; therefore, they are useful but not the optimal objective function to guide the search for a signaling pathway.

In this study, we propose a novel signal-oriented framework for searching cancer pathways by combining gene expression with SGA data. The premise underlying our approach is as follows: since the state of a signaling pathway can be reflected by the expression state of a set of genes it regulates (i.e., its signature), the task of searching for a pathway can be formulated as a search for a set of SGAs that collectively exhibit strong information with the state of a gene expression signature. Under such a setting, mutual exclusivity and co-expression of SGA-affected genes can further be used as auxiliary objective functions to constrain the search space and to enhance the confidence of the results. This approach addresses the fundamental task of the pathway discovery—finding a set of SGAs perturbing a common signal.

We applied this novel framework to the ovarian cancer and glioblastoma data from TCGA, followed by systematically evaluating the impact of the signal-oriented approach on the search for driving pathways and comparing the performance of our exact algorithm with that of heuristic or stochastic algorithms. We show that the signal-oriented approach provides a general framework in which different pathway-searching algorithms can be combined with different signal-oriented objective functions (beyond those discussed here) to explore new directions for studying cellular signaling systems.

## Materials and Methods

**Fig 2:**
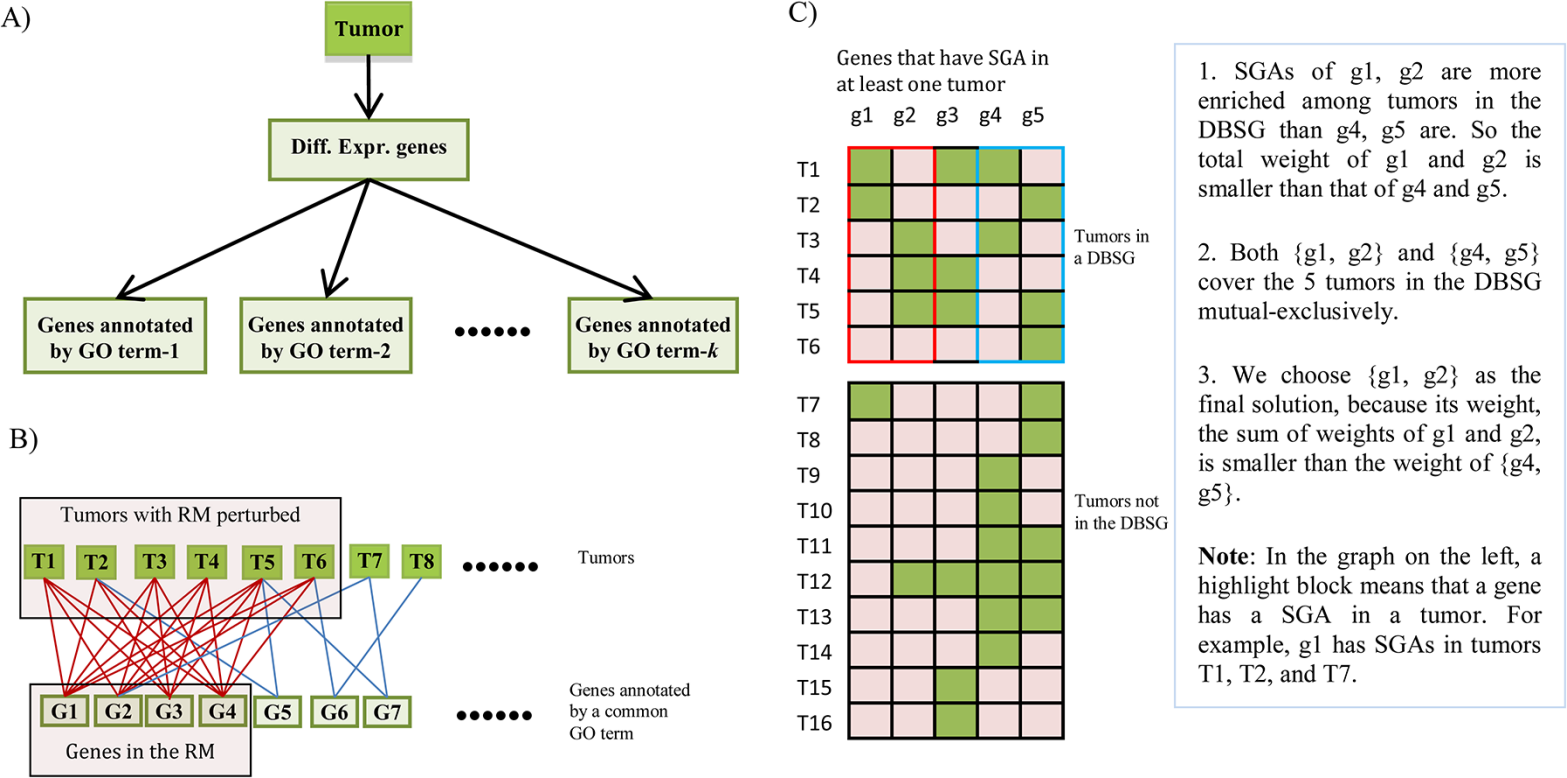
Overall scheme. **A**) Use GO structure and semantic information to categorize differentially expressed genes in each tumor into functionally coherent subgroups. **B**) Find a dense bipartite sub-graph (DBSG) consisting of a subset of genes and a subset of tumors, such that the genes in the DBSG form a response module (an RM) to a perturbed signal in the tumors. **C**) Search for SGAs perturbing a signal regulating an RM. First, assess the strength of the associations (the enrichment weight) between SGA-affected genes and an RM. Then find a set of SGAs that are mutually exclusive and that cover a maximum number of tumors in the DBSG; minimize the weight sum of the SGAs in the solution, and you have the *weighted mutually exclusive maximum set cover* problem. Note that SGA-affected genes are denoted with the lower case, while differentially expressed genes are rendered in the upper case.

The overall framework of this study is shown in Fig 2, which consists of the following steps to search for a set of mutually exclusive driver SGAs that affect a common signal: 1) We first identified differentially expressed genes from each tumor, and then grouped genes into non-disjoint functional sets according to their Gene Ontology[18] (GO) annotation using the methods previous developed by our group [19–21], such that functions of the genes in a set are coherently related to each other and are summarized by a GO term from the Biological Process Domain of the GO (Fig 2A). 2) After selecting the differentially expressed genes annotated with a common GO term across tumors, we constructed a bipartite graph consisting of tumors on one side and the genes on the other side, and we then searched for a densely connected subgraph (Fig 2B). We hypothesized that, if a set of genes that are coherently related (sharing a common GO annotation) and co-differentially expressed in multiple tumors, they are likely regulated by a common signal that is perturbed in these tumors. Thus, the genes constitute a gene expression module responding to the signal. We refer to such a module as a “response module” (RM). 3) We then designed an exact algorithm to search for a set of SGAs that are significantly enriched in the tumors in which an RM was perturbed, with the constraint that the SGAs in the solution set exhibited a mutually exclusive pattern among tumors in the RM (Fig 2C).

### Data pre-processing

Data on somatic mutation, copy number alteration, and gene expression from 568 ovarian cancer tumors and 513 glioblastoma tumors, as well as 8 normal control samples from ovarian tissue and 10 cases of normal brain samples were downloaded from the TCGA[4,5]. For each tumor, we considered a gene as being differentially expressed if its expression value increased or decreased at least 3-fold in comparison to the median value of the gene in the control samples. We defined a gene as affected by an SGA event if it had a non-synonymous single nucleotide variation in its coding region, and/or an insertion or a deletion; we also labeled a gene as affected by an SGA event if it had copy number alteration (with the GISTIC[22] score ≥ 2 and z-score ≥ 1.64 or GISTIC score ≤ -2 and z-score ≤ -1.64, where the z-score is obtained by the z-transformation of the expressions of the gene across all tumors). Hence, only the copy number alterations that affected gene expression (with a *p*-value of 0.05) are included. We removed the genes that exhibit both amplifications and deletions (with the smaller fraction being over 10%) in a given set of tumors that are supposed to have a common signal perturbed as inconsistent genes. For example, if a gene *X* is affected by copy alteration, where 85% of events are amplification and 15% are deletion (thus smaller fraction is over 10%), we would remove this gene from the consideration. This is a relatively conservative consideration of those genes with consistent copy number alteration direction as potential drivers. Finally, if two genes exhibit perfect correlation (co-amplified or co-deleted whenever altered), we treat these genes as one common genomic alteration. These procedures lead to a tumor-by-gene binary matrix recording differentially expressed genes, and a tumor-by-gene binary matrix recording SGA events in tumors.

### Identifying gene modules as signal-response units

To deconvolute signals embedded in the gene expression data, we hypothesized that if a set of genes performs coherently related functions and tends to be co-regulated in multiple tumors, the genes are likely regulated by a common signaling pathway as a module. To find such modules among the cancer tumors, we employed a knowledge-driven data mining approach, developed in our previous studies[19–21], which consists of two major procedures: 1) identifying functionally coherent gene subsets among the differentially expressed genes in each tumor, such that each gene subset is annotated by a GO term that summarizes the function of the genes; and 2) further identifying the gene subsets that are differentially expressed in multiple tumors, which is formulated as the dense bipartite subgraph finding problem. Researchers have used set cover[23,24] or the extension of the set cover – module cover [25] model to find gene subsets that are differentially expressed because of pathway perturbation. Our two-step method differs from these in that genes included in each solution subset are both functionally coherent and co-expressed in a considerable number of tumors. We also obtained the tumors that perturb the pathways regulating the expression of each gene subset.

To identify functionally coherent gene subsets, we first found a tumor’s differentially expressed genes and grouped them into non-disjoint subsets by mining their annotations [19–21]. This was achieved by representing the hierarchical structure of GO terms as a directed acyclic graph and searching for genes annotated with closely related GO terms. We first associated genes to the GO terms according to annotations of the genes. We then iteratively merged highly specific GO terms and their associated genes to their parent GO terms according to a procedure [19] that strives to minimize the loss of semantic information during the process. In this fashion, we can group genes annotated with closely related terms into a set annotated with a more general GO term that retains the information of the original annotations. We stop the procedure if a further merge leads to a non-coherent gene set, according to a quantitative metric that assesses the statistical significance of functional coherence of the gene set [19,21]. This procedure enabled us to partition differentially expressed genes from each tumor into non-disjoint, functionally coherent subsets.

Next, we further identified the functionally coherent gene subsets that are affected in multiple tumors. We modeled this problem as a **d**ense **b**ipartite **s**ub**g**raph (DBSG) finding problem, of which the detailed algorithm was introduced in our previous work[21]. Briefly, we pooled gene subsets annotated with a common GO concept across all tumors and constructed a bipartite graph, in which the vertices on one side represent the pool of differentially expressed genes sharing the GO annotation, and the vertices on the other side represent the tumors; an edge between a gene and a tumor indicates that the gene is differentially expressed in the tumor. We then searched for a subset of genes that are co-differentially expressed in multiple tumors. We formulated our task as follows: find a maximum dense bipartite subgraph such that each gene must be connected to at least 75% (a connectivity ratio) of all tumors in the subgraph and each tumor must be connected to at least 75% of all genes in the subgraph. Thus, each DBSG consists of a set of genes, i.e., an RM, and a set of tumors in which the RM is perturbed.

### An exact algorithm for finding a minimum-weight, mutually exclusive set

To search for the candidate members of a signaling pathway regulating an RM, we aimed to find a set of SGA-affected genes that has the following properties: 1) the SGA events affecting the genes cover as many as possible of the tumors in which the RM of interest is perturbed; 2) the SGA events carry strong information with respect to the expression state of an RM, or, in other words, the SGAs are significantly enriched in tumors in which RMs have been perturbed; and 3) each tumor is covered by only one gene in the solution set (thus mutually exclusive. Note: SGA events are only mutually exclusive among tumors in each DBSG). By assigning a weight to each SGA to reflect the amount of information the SGA carries with respect to the state of the RM, we formulate this computational problem as the *weighted mutually exclusive maximum set cover* problem, a variant of the well-known *set cover* problem in algorithm theory[26].

To assess the strength of association of an SGA-affected gene (a genome locus) with the state of an RM, we apply a hypergeometric test to compute the enrichment of the SGA events of the gene in tumors within which the RM has been perturbed[27]. We then set the log *p*-value of the SGA enrichment analysis as the weight for the gene; thus, a set of SGAs with a smaller total weight tends to carry more information with respect to the RM when compared to another gene set with a greater total weight.

Fig 2C illustrates the problem setting as follows. Of the 16 tumors and 5 genes in a dataset (Fig 2C), 6 tumors are included in a DBSG. We define that a tumor is covered by a gene if an SGA affecting the gene occurs in the tumor; we then represent each gene by the subset of tumors in the DBSG it covers. In our example, g1 = {T1, T2}, g2 = {T3, T4, T5}, g3 = {T1, T4, T5}, g4 = {T1, T3}, and g5 = {T2, T5, T6}. For each DBSG, we define the set of all tumors in the DBSG, X, as the ground set; for example, in the figure, *X* = {T1, T2, T3, T4, T5, T6}. We define F as the set of the candidate genes; in our example, F = {g1, g2, g3, g4, g5} = {{T1, T2}, {T3, T4, T5}, {T1, T4, T5}, {T1, T3}, and {T2, T5, T6}}. We define *w*: F→ (-∞, ∞); the function *w* gives weight to each gene. Given a subset of genes, F’⊂F, if no two elements in F’ have any common element, i.e., if no two genes cover the same tumor, we then say that F’ is mutually exclusive; the weight of F’ is ${\sum_{S}}_{\in F’}$ *w*(*s*). The problem’s goal is to find a mutually exclusive subset of F that covers a maximum number of elements of *X* (i.e., that covers a maximum number of tumors). If we find two or more solutions, e.g., {g1, g2} and {g4, g5}, that cover the maximum number of elements, we choose the solution with the minimum weight, {g1, g2}. This is the formal definition of the *weighted mutually exclusive maximum set cover* problem.

As is the case with the formulations of other studies on mutual exclusivity[12–14,16,17], our problem is NP-hard (see the proof in a separate technical report[28]). Previous studies used heuristic or stochastic algorithms [12–14,16,17] to handle the mutually exclusive set cover problem or its variants, but such algorithms do not guarantee the finding of optimal solutions. In this study, we developed an exact algorithm, called the Weighted **M**utually **E**xclusive Maximum Set Cover algorithm (or the **ME** algorithm), that guarantees the finding of exact optimal solutions. The algorithm uses parameterized techniques[29], such that the running time is an exponential function of a parameter that can be bounded by a small number for certain specific applications, instead of the exponential functions of large input sizes that are generally used for solving general NP-hard problems. Because our problem involves about 600 tumors and 30,000 genes, an exponential function of any of these input sizes would be intractable. However, using the parameterized technique, we developed an algorithm whose worst time complexity is O*(1.325^*m*^), where *m*, the parameter, is the number of candidate genes when we search driver SGAs affecting a pathway. In fact, the actual running time of the algorithm is much smaller than that of the worst time complexity; the algorithm can finish our computation task on a workstation in 5 to 10 minutes, even when *m* is 200, which is sufficiently large candidate size for searching driver SGAs that perturb a signaling pathway. We refer to a set of solution SGAs as perturbation module (PM) to reflect that they may perturb a common signaling pathway.

Our method adopts a branch-and-bound principle: the algorithm first finds a subset in F, and then branches on it. Due to the mutual exclusivity constraint, if any two subsets in F overlap, then at most only one of them can be chosen into the solution. For example, suppose that the subset *S* intersects with other *d* subsets in F; then, if *S* is included into the solution, *S* and other *d* subsets intersecting with *S* will be removed from the problem, and if *S* is excluded from the solution, *S* will be removed from the problem. We continue this process until the resulting sub-problems can be solved in constant or polynomial time. Let *T*(*m*) be the number of computations needed when call the algorithm with *m* subsets in F, then we can obtain the recurrence relation *T*(*m*) ≤ *T*(*m*−(*d*+1))+*T*(*m*−1). As if *d* = 0 for all subsets in F, the problem can be solved in polynomial time (all subsets in F will be included into the solution), in the recurrence relation, *d* ≥ 1. Therefore, we can obtain *T*(*m*) ≤ 1.619^*m*^, which means the problem can be solved in O*(1.619^*m*^) time (**note:** Given the recurrence relation *T*(*k*) ≤ ∑_0 ≤ *i* ≤ *k*-1_ *c*_*i*_ *T*(*i*) such that all *c*_*i*_ are nonnegative real numbers, ∑_0 ≤ *i* ≤ *k*-1_ *c*_*i*_ > 0, and *T*(0) represents the leaves, then *T*(*k*) ≤ *r* ^*k*^, where *r* is the unique positive root of the characteristic equation *t* ^*k*^- ∑_0 ≤ *i* ≤ *k*-1_ *c*_*i*_ *t* ^*i*^ = 0 deduced from the recurrence relation[30]). We improved the algorithm’s running time by carefully selecting subsets in F for branching. As the proof of the algorithm is very involved, we present the details in the technical report[28].

We have implemented all algorithms for the paper. Supplement results and codes for algorithms can be found at: http://pitttransmed-tcga.dbmi.pitt.edu/mutuallyExclusive/.

## Results

### Identify RMs to reveal perturbed cellular signals

Using the integrated knowledge-mining and data-mining approaches, we identified 88 dense bipartite subgraphs (DBSGs) from the ovarian cancer tumors. Each DBSG includes a response module (RM) consisting of at least 10 genes that are differentially expressed in 30 or more tumors. Based on our functional coherence analysis, the genes in an RM were functionally related to each other. To further corroborate these results, we also evaluated the RMs using the Ingenuity Pathway Analysis (IPA— http://www.ingenuity.com/); each of our RMs was found to significantly overlap with at least one of the IPA networks. For example, we found 55 RMs, of which more than 90% of their genes overlapped with at least one network from the Ingenuity network database (results are presented in a supplementary website so that researchers can browse the RMs and their driver SGAs).

**Fig 3:**
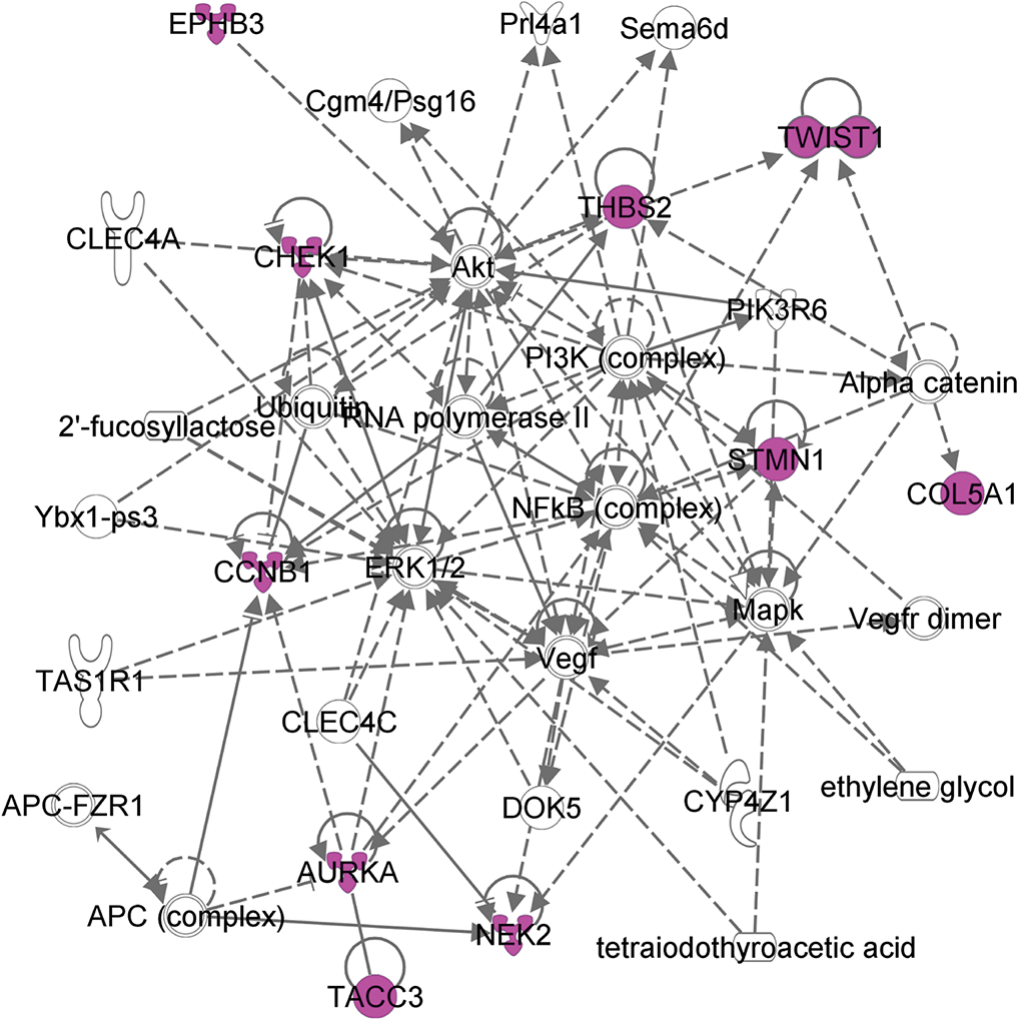
Overlapping of genes in RM U_GO0051128 with an IPA network. A total of 10 out of 11 genes in the RM overlap with an IPA network (highlighted).

As an example, an RM that consists of 11 genes (*AURKA*, *CCNB1*, *CHEK1*, *COL5A1*, *EPHB3*, *NEK2*, *PSRC1*, *STMN1*, *TACC3*, *THBS2*, *TWIST1*) that are up-regulated in 62 tumors. The biological processes in which the genes are involved are summarized by the GO term GO:0051128: *Regulation of Cellular Component Organization* (note: because genes in each RM are annotated by a GO term, we use GO term IDs to name RMs and PMs; we also use U_ or D_ to indicate whether genes in the RM are up-regulated or down-regulated, respectively). For example, the designation “RM U_GO0051128” means that the genes in the RM are up-regulated and that they are annotated by the GO term GO:0051128; “PM D_GO0009611” is the PM that regulates the RM D_GO0009611). We found that 10 of those 11 genes are in an IPA network (Fig 3) that is labeled as “*Cellular Assembly and Organization*, *Cellular Function and Maintenance*, *Cell Morphology*”. Previous laboratory studies show that 9 of these genes play important roles in tumor initiation and progression in different types of cancers. For example, *AURKA* was found overexpressed in the early stage ovarian tumors, therefore suggesting that the alteration of *AURKA* could be an early event of ovarian cancer[31]. High levels of *AURKA* expression is closely correlated to poor survival of patients with ovarian cancer [32]. The proliferation-related targets *AURKA* and *CCNB1* were overexpressed in clinical ovarian tumor specimens[33]. Our predicted results corroborate with the established roles of *AURKA* and *CCNB1* in cancers. In addition, many references also show that overexpression of seven of the remaining nine genes in the RM are related to cancers (S1 Table).

Another example of a cancer-related RM, annotated with the GO term GO:0010564 (*Regulation of Cell Cycle Process*), includes 10 genes (*BIRC5*, *CCNB2*, *CDC7*, *CDKN2A*, *CENPE*, *CENPF*, *CHEK1*, *NEK2*, *TIMELESS*, *UBE2C*) that are up-regulated in 140 tumors. All of those 10 genes are in an IPA network related to “*Cell Cycle*, *DNA Replication*, *Recombination*, *and Repair*, *Cellular Assembly and Organization*” (see Supplement). A literature search shows that 9 of 10 genes in the module are related to several types of cancers (S2 Table). Among them, expression of *CENPE* and *CCNB2* correlates with worse clinical outcomes of patients with breast or ovarian cancers [34–36]. The fact that the genes in these RMs are functionally coherently related and co-regulated in multiple tumors from different types of tumors indicates that their aberrant expression is likely regulated by a common signal; thus, expression state of an RM can be utilized as the readout of the state of a hidden signal, allowing the search for the SGA events perturbing the signal. One interesting observation in this RM is that the *CDKN2A*, a tumor suppressor, is overexpressed. By checking the copy number alteration data, we found that the overexpression of *CDKN2A* in most of 140 tumors were not caused by the gene amplification. The similar phenomenon was observed in large number of tumors in other tumor types, such as GBM and HNSC, where the *CDKN2A* was overexpressed in almost all tumors without *CDKN2A* amplifications. The explanation of this phenomenon needs the further study from cancer biologists.

This framework was also applied to TCGA data of glioblastoma multiform (GBM), the most malignant cancer in the brain, and we identified 101 RMs. Comparing RMs in GBM with ones in ovarian cancer, 38 RM pairs were annotated by an identical GO term in both ovarian cancer and GBM, among which 17 modules are significantly overlapped (*p*-value and *q*-value of overlapping < 10^–4^, S3 Table). For example, the RMs annotated with U_GO0007067 (*mitotic nuclear division*) found from GBM and ovarian cancer have 18 and 17 genes respectively, in which 15 genes are in common, and the union of the two RMs includes 20 genes. As expected, literature studies indicate that almost all the above genes and most of other significant overlapping RMs are related to cancers (S4 Table), including those involved in U_GO0009611 (*response to wounding*) and U_GO0006974 (*cellular response to DNA damage stimulus*). Thus the approach of searching RMs as reflections of perturbed cellular signals is generalizable to different types of cancers and capable of finding cancer-related RMs.

We further investigated if the expression states of RMs are relevant to patients’ clinical outcomes, we dichotomized GBM patients from TCGA according to the expression state of each RM, followed by survival analysis. We found the expression states of 25 RMs are associated with significant differences in patients’ survival (p-values < 0.05 and q-values < 0.05 for Kaplan-Meier analysis, see S5 Table and S1 Fig). We also applied these methods to the breast cancers from TCGA for searching RMs (data not shown). We used the breast cancer RMs as features for predicting survival of the patients studied by Curtis et al [37] in an open research challenge (the DREAM 7 Challenge), in which RMs were found to be highly predictive of patient survival [38]. Therefore, the expression states of RMs reflect the states of cancer cells, which is determined by cellular signal transduction pathways.

### Finding SGAs underlying RMs

We hypothesized that the differential expression of an RM in a tumor is due to the aberrant signal resulting from pathway perturbation by at least one of the SGAs (somatic genome alterations) observed in the tumor. Since somatic copy number alterations are common in ovarian cancers (which may contribute to differential expression of genes), we first examined if identified RMs are driven by copy number alterations. For each RM, we treat differential expression of a gene in a tumor as a differential expression event. We also define that it is driven by a copy number alteration event if the gene is copy number altered in the tumor. We then calculated the fraction of copy-number-alteration-driven differential expression events for each module and averaged them across the RMs, which shows that, on average, only 3.4% of differential expression events are likely driven by copy number alteration. The results support our hypothesis that the differential expression of an RM is driven by a pathway rather than by direct copy number alterations. As such, a reasonable strategy for identifying the signaling pathway regulating the RM is to pool the tumors in which the RM is differentially expressed and further search for a subset of the SGA events in these tumors that carries the strongest information with respect to the expression state of the RM. We refer to a module of genes affected by such SGAs as a “perturbation module” (PM).

When given a DBSG, we first identified all SGA events observed in the tumors within it, and then calculated enrichment of SGAs affecting a gene using a hypergeometric test, assigning the enrichment *p*-value as the weight of the gene. We applied the ME algorithm to identify an optimal PM for each DBSG, using up to 200 SGAs with the lowest *p*-values as input, a sufficiently large number when considering that most known biological pathways contain around tens of proteins. The sizes of the returned PMs ranged from 3 to 14 genes, with an average of 7.14. Since our algorithm strives to include SGAs that are specifically enriched in the tumors in a DBSG, the genes in a PM as a whole are highly enriched in the tumors in a DBSG, with enrichment *p*-values ranging from 5.68×10^−4^ to 7.16×10^−25^ (median: 8.97×10^−14^).

Since SGA events are in effect randomized perturbatons performed by nature, a strong correlation between SGA events and the expression state of an RM suggests that SGAs influence gene expression rather than the reverse direction (differential gene expression causing SGAs). Thus, genes in each PM identified in our study are likely members of the signaling pathway perturbed in tumors that underlie the differential expression of the genes in an RM. Though experimental validation of the results could be conclusive, it is extremely costly. Therefore, we validated our results by comparing them to the existing knowledge using the IPA package, with the understanding that while the knowledge base of the IPA may be incomplete, it is, nonetheless, an accessible approach. Our findings indicated that most PMs were significantly associated with different diseases and/or disorders (65 PMs with both *p*-values and *q*-values of at most 0.001, with a median of 9.21×10^−4^); among them, 30 PMs were related to cancers with both *p*-values and *q*-values smaller than 0.001. We further investigated whether the identified PMs could be mapped to known signaling pathways, concluding that, indeed, many PMs were enriched in known pathways, including 51 PMs that were enriched in a known pathway with both *p*-values and *q*-values of at most 0.01.

**Fig 4:**
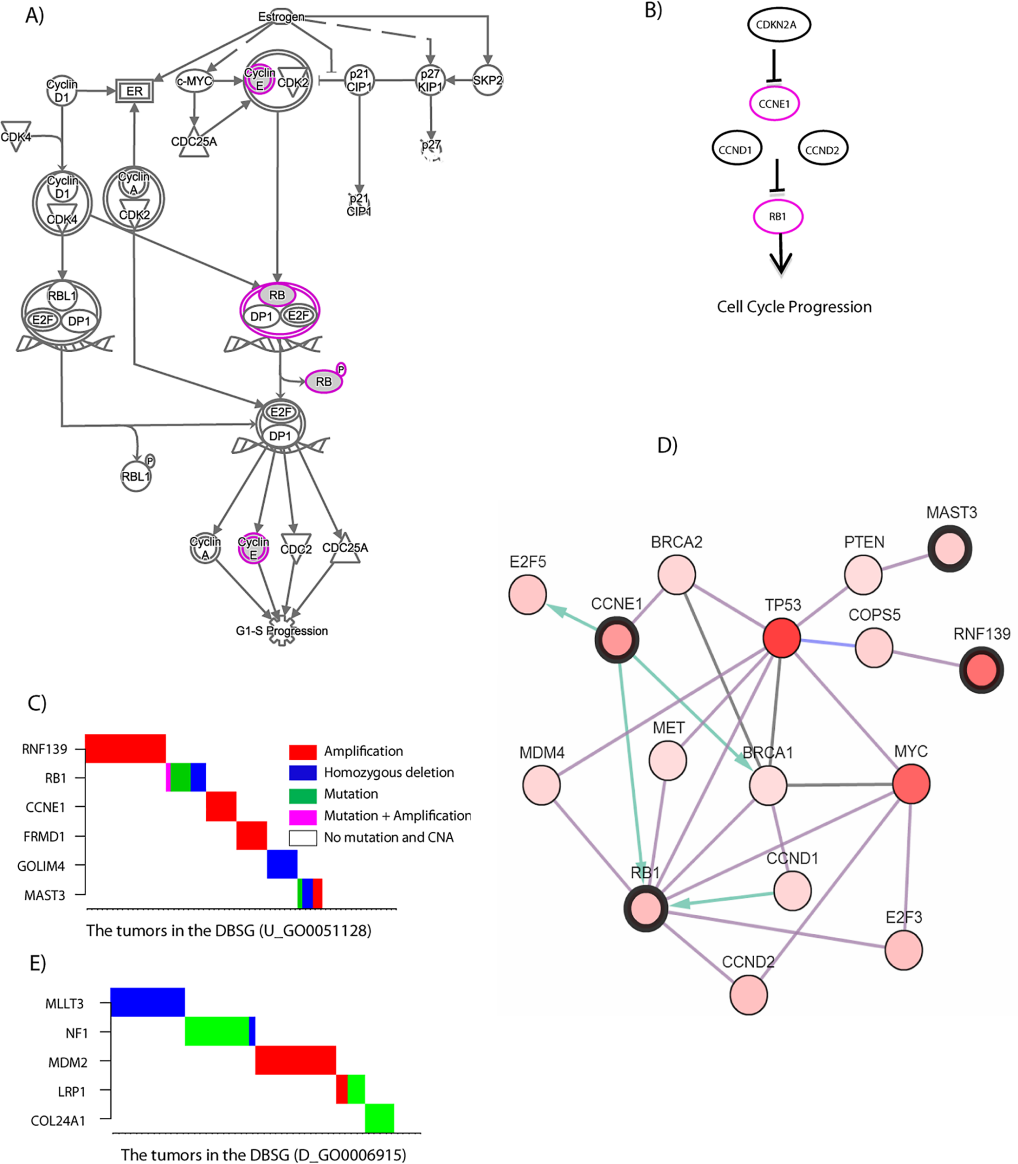
The PM corresponding to RM U_GO0051128. **A**) Overlap between the PM U_GO0051128 and a known pathway. **B**) Overlap of the PM U_GO0051128 with the RB pathway. **C**) SGA pattern of the PM U_GO0051128. **D**) Genes in the PM U_GO0051128 closely interacting with other oncogenes. **E**) SGA pattern of the PM D_GO0006915.

As an example, we examined the PM corresponding to the RM studied in the previous section, U_GO0051128, which consists of 6 genes (*CCNE1*, *RB1*, *FRMD1*, *COLIM4*, *MAST3*, *RNF139*). The genes in the PM are enriched in the IPA pathway “Estrogen-mediated S-phase Entry” (Fig 4A), with a *p*-value of 1.83×10^–5^. This PM has two genes (*RB1* and *CCNE1*) in the well-characterized RB1 cancer pathway that plays important roles in ovarian cancer tumorigenesis (Fig 4B) [5]. Golgi integral membrane protein 4 (*GOLIM4*) is a type II Golgi-resident protein that involves in processing proteins synthesized in ER and assist in the transport of protein cargo through the Golgi apparatus[39]. These transported proteins include ones shown in Fig 4A. Ring finger protein 139 (*RNF139*) is a multi-membrane spanning protein with ubiquitin ligase activity. RNF139 interacts with tumor suppressor VHL and JAB1[40], the latter is responsible for the degradation of tumor suppressor *CDKB1B*/p27CIP1 in this pathway (Fig 4A). *GOLIM4 and RNF139* in this PM were found as potential cancer drivers in various types of cancers [41–43]. Additionally, *MAST3*, *RB1*, and *CCNE1* in this PM are critical in regulating cell cycle [44–46]. As shown in Fig 4C, the mutually exclusive pattern of the SGAs affects genes in this PM identified from the 62 tumors in which this RM was perturbed. It is of highly significance that our algorithm predicts that the amplification of *CCNE1* gene conveys the identical information as to the mutation or deletion of *RB1*. As shown in Fig 4B, the protein encoded by *CCNE1* inhibits that of *RB1* (Fig 4B); both amplification of *CCNE1* and mutation/deletion of *RB1* have the same effect on a common signal, leading to aberrant regulation of cell cycle entry and thereby causing cancers. Indeed, 6 out of 11 genes in the RM U_GO0051128 are related to cell cycle. When searching the PASTAA (http://trap.molgen.mpg.de/PASTAA.htm) database for enriched transcription factor binding sites of genes in this RM, the binding site of *E2F1* (a transcription factor directly downstream of *RB1*) was the most significantly enriched region in the promoters of the genes in this RM (S6 Table). Thus, our algorithm correctly identified a perturbed signaling pathway and its downstream target genes. Fig 4D further shows that proteins encoded by the genes in the PM U_GO0051128 directly interact with other well-known oncogenes, such as *TP53*, *MDM4*, *CCND1*, *CCND2*, *MYC*, *E2F3*, *E2F5*, *BRCA2*, *PTEN*, *MET*, *and COPS5* (S7 Table). Thus, the results indicate that the signal-oriented approach leads to biologically sensible findings. However, a challenging issue of handling copy number alteration is that a set of genes can be co-amplified (co-deleted) within highly overlapping but not perfectly identical copy number alteration fragments in different tumors. Thus, it would be difficult to differentiate the signals of such alterations. For example, *RNF139*, *TRMT12*, *ZNF572*, *SQLE*, *MYC* and other genes are often co-amplified but not perfectly correlated in various types of cancers including ovarian cancer(S2 Fig), among which *MYC* is a known cancer driver gene in numerous types of cancers[47]. When examining other genes in this region, we found that certain amplification events were not associated with corresponding gene expression changes, hence disqualifying these alterations as a potential driving event. Our algorithm returned *RNF139* in the solution because the perturbation events of *RNF139* (copy number alteration of a gene with associated expression change) had strongest information with respect to this RM. While the signal of *RNF139* may be convoluted with those of the other genes in the fragment, there is evidence that *RNF139* contributes to cancer development by regulating tumor suppressors p27 and VHL[40] that are both critical in cancer development[48,49]. Our results indicate that our methods are capable of identifying the copy number alteration fragment that has strong information with respect to an RM as well as finding the most representative gene. The latter is closely related to the capacity of finding the peak of a commonly amplified or deleted region[50]. However, determination of whether this representative gene is an authentic driver requires thorough interrogation of neighboring genes followed by experimental validation.

Next, we examined another perturbation module PM D_GO0006915 obtained from TCGA GBM data. The module has five genes *MLLT3*, *NF1*, *MDM2*, *LRP1*, and *COL24A1*. *MLLT3*, *NF1*, and *COL24A1* are either deleted or mutated while *MDM2* is amplified in tumors (Fig 4E). We found that the genomic alterations of these genes suppress the expressions of a set of genes related to *apoptotic process of cells*. The *MDM2* is a well-known oncogene that inhibits tumor suppressor p53 function [51–53]. Inhibition of *MDM2* induces cell apoptosis [54,55] and reactivates p53 in GBM cells, resulting in inhibition of GBM cell growth in vitro and in GBM xenografts in mice[56]. Thus, it is plausible that amplification of *MDM2 in* the module PM D_GO0006915 will inhibit cell apoptosis. Inactivation of *NF1* by germline mutations are predisposed to the development of benign and malignant tumors of peripheral and central nervous system including GBMs[57,58]. Loss of NF1 also renders cancer cell resistant to apoptosis [59]. On the other hand, forced expression of *MLLT3*, which is deleted in the module PM D_GO0006915, significantly increased cell apoptosis [60]. Lastly, low—density receptor protein 1 (LRP1) activates Akt pro-survival pathway thereby inhibit cell apoptosis in neurons[61]. Depletion of *LRP1* led to an increase in cell apoptosis [61,62], thus corroborating with our findings that amplification of *LRP1* inhibits apoptosis. Since *LRP1* mutations have not been reported in cancers, we predict that these mutations exert a similar effect as their gene amplification events. Taken together, our identification of alterations in this PM in GBM validated the established functions of these oncogenic drivers in tumor initiation and progression in GBMs and other types of cancers.

### Systematic evaluation of the impact of the signal-oriented approach and use of the exact algorithm on finding PMs

Our framework employed two main innovations. First, we revealed the perturbed cell signals from each tumor, and we utilized the information to search PMs in a signal-oriented fashion. Second, we developed an exact algorithm (the ME algorithm) that efficiently solves the weighted mutually exclusive maximum set cover problem. In this section, we systematically evaluate the impact of the above approaches on revealing cellular signals and identifying the pathways transmitting them.

#### 1. Evaluation of the impact of the signal-oriented approach on finding PMs

In this subsection, we assess the impact of signal-oriented approach for identifying tumors with a common signal perturbed, i.e., identifying tumors in which a common RM is perturbed, on the quality of PMs from an information-theory point of view. Since it is uncommon that a signaling pathway is universally perturbed in all tumors, a set of SGAs in a PM randomly distributed among tumors is less likely to affect a specific cellular signal. On the other hand, if the SGAs in a PM are specifically enriched in a subset of tumors, this PM has high information content with respect to the input tumors, potentially reflecting a common characteristic shared by the tumors, such as a common perturbed pathway. Imagine that two methods, A and B, are repeatedly used to dichotomize tumors into two subsets, and that a common algorithm is then employed to search for a PM covering one of them as input. If method A consistently yields an input set that leads to more informative PMs in comparison to method B, we say that method A reveals specific information in the tumors. This enables us to determine if our method of finding a set of tumors sharing a common RM enhances the capability of finding informative PMs.

Based on the above assumptions, we used a well-established program, the De novo Driver Exclusivity (Dendrix [16]), as an unbiased tool to search for SGAs that cover a set of tumors with strong mutual exclusivity, in order to assess whether dichotomizing tumors based the expression states of an RM reveals biological information. Dendrix, developed in a seminal study by Vandin et al. [16], is a program that searches for driver mutations in a pathway (i.e., a PM) by exploiting the mutually exclusive property of SGAs. Given a set of tumors and the SGAs observed in the tumors, its task is to find a PM of size *k—*a parameter to be assigned by a user—that maximizes the coverage of input tumors while minimizing the overlapping coverage of tumors by SGAs (thus maximizing mutual exclusivity). Due to the NP-hard nature of the problem, the Dendrix utilizes a Monte Carlo Markov chain approach to address the task. Using a set of tumors and their SGAs as input, Dendix returns a collection of SGA sets of size *k* as candidate PMs, and it assigns a weight to each PM to reflect the PM’s quality (the higher, the better); this is referred to as the *D-weight* score in the following discussion.

**Fig 5:**
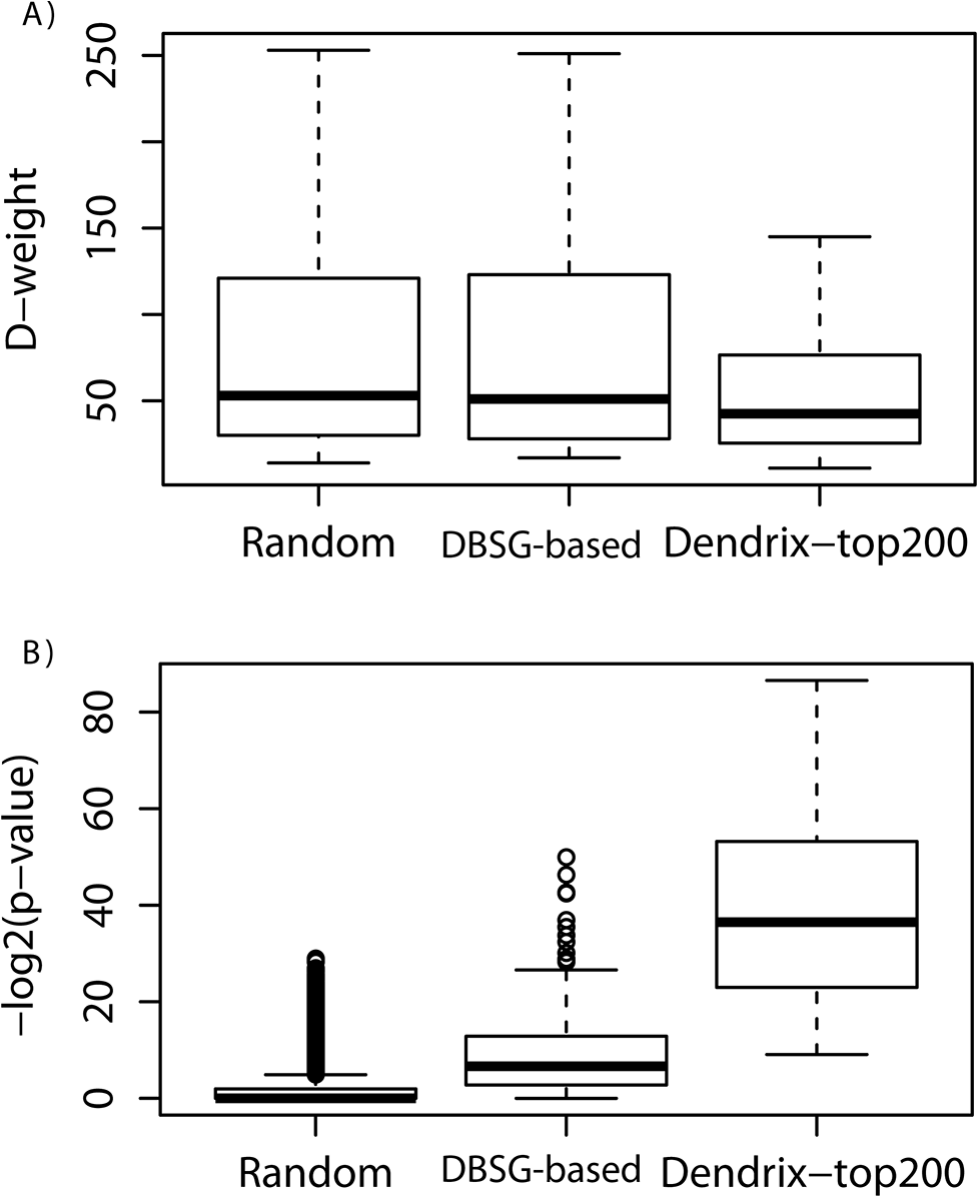
The impact of the signal-oriented approach on the qualities of PMs. **A**) Distributions of D-weight. **B**) Distributions of enrichment *p*-values. (Note: D-weight – the solution weight defined by Dendrix, where a solution with high coverage (of tumors) and low overlap will have a high score. The Enrichment *p*-value – the solution weight defined by our method, where a solution whose somatic mutation and copy number alteration are enriched in tumors that perturb a common signal will have a good *p*-value.)

We applied Dendrix to the tumor set from each of the 88 DBSGs, we also applied Dendrix to randomly drew 88 tumor sets and with matching sizes (repeated 128 times). In this way, the DBSG-based input utilized the information that common pathway may be perturbed in the input tumors (due to the fact that an RM is differentially expressed in them), whereas the random tumor sets did not utilized such biological information; we refer to the derived PMs as *DBSG-based* and *random*, respectively, in the following discussion. From each input tumor set, we specified the parameter *k* to be the same size as the solution PM returned by our ME method on the same input, and we selected the solution PM with the best D-weight score in the following analyses. The box plots in Fig 5A illustrate the distribution of the D-weight scores derived from both the DBSG-based and the random tumor sets. The results indicate that Dendrix is capable of finding PMs from both DBSG-based and random tumor sets that have similar coverage and mutual exclusivity. Note that the action of searching for a DBSG and drawing a random tumor set *de facto* dichotomized tumors into two groups: input tumors and the rest. Thus, we were able to calculate the enrichment of the solution SGAs within the input tumor set (DBSG-based or random) versus that of the rest of the tumors, using a hypergeometric distribution test [27] to assess the quality of returned PMs. Fig 5B shows that the PMs from the DBSGs are significantly enriched in the input tumors, with a mean of log (base 2) *p*-values of −11.11; on the other hand, the enrichment *p*-values derived from the random tumor sets are much less significant, with a mean of log *p*-values of −1.92 (which corresponds to a *p*-value of 0.26, a non-significant *p*-value).

The results described above indicate that the expression states of an RM reflect real biological signals shared by a set of tumors, thus enabling Dendrix to identify the candidate driver SGAs that are strongly associated with specific input tumors. On the other hand, feeding Dendrix with tumors with no specific information leads to the discovery of uninformative SGAs. The striking difference is solely due to different inputs, because Dendrix was able to find mutually exclusive SGA sets that provided equal coverage of tumors from random tumor sets. This fact, in turn, suggests another important conclusion: that the mutual exclusivity property of a set of SGAs alone is not sufficient to indicate that the SGAs carry specific information; therefore, it should not be the sole objective function to guide the search for signaling pathways.

We further assessed if the Dendrix program could also benefit from the information of RMs in a quantitative fashion, where such information was provided to the program in the form of constrained inputs. We ranked SGAs in an ascending order based on the p-value of their enrichment in tumors aberrantly expressing an RM. As such, the smaller the p-value, the stronger an SGA is associated with the expression state of an RM. Therefore, from an information viewpoint, the more information the SGA carries with respect to the RM. It should be noted that the most frequent SGA event, TP53 mutations, carries very low amount information with respect to most RMs. We then use the top 200 SGAs as input for the Dendrix program, which is equivalent to allowing the program to take advantage of the information with respect to an RM to search a pathway in a signal-oriented fashion. We refer to PMs derived from this experiment as the *Dendrix-top200*. When compared to the results from DBSG-based tumor sets without constraints, the PMs from the Dendrix-top200 experiment were significantly more enriched in the input tumors, with an average log *p*-value of −38.24, indicating that the signal-oriented approach indeed enhances the ability to identify informative SGAs; thus, they are more likely to be biologically sensible candidates for cancer pathways. Our study in using Dendrix to search for more informative candidates reflects the generalizability of our signal-oriented approach.

#### 2. The performance of our exact algorithm

In this study, we designed an exact algorithm to find an optimal PM by pursuing three objectives: 1) that the PM covers the maximal number of input tumors; 2) that the PM covers the maximal number of input tumors with a minimum total weight; and 3) that each tumor is covered by at most one gene in the solution. Unlike greedy or stochastic algorithms (e.g., the Dendrix), an exact algorithm guarantees the finding of an optimal solution, and thus should out-perform heuristic algorithms. To illustrate this advantage, we compared the performance of our ME algorithm with that of the Dendrix’s program, when both were applied to the 88 DBSGs derived from the TCGA ovarian cancer data.

In order to compare the programs under similar circumstances, we constrained the input SGA-affected genes to conform to those used in the Dendrix-top200 experiment; we then set the weight for all input genes to 1 and applied the Dendrix and the ME programs to the data. We denoted the results from this experiment as “ME-uw,” and we analyzed the enrichment of PMs in input tumor sets as well as the PMs’ D-weight scores in order to compare the returned results.

**Fig 6:**
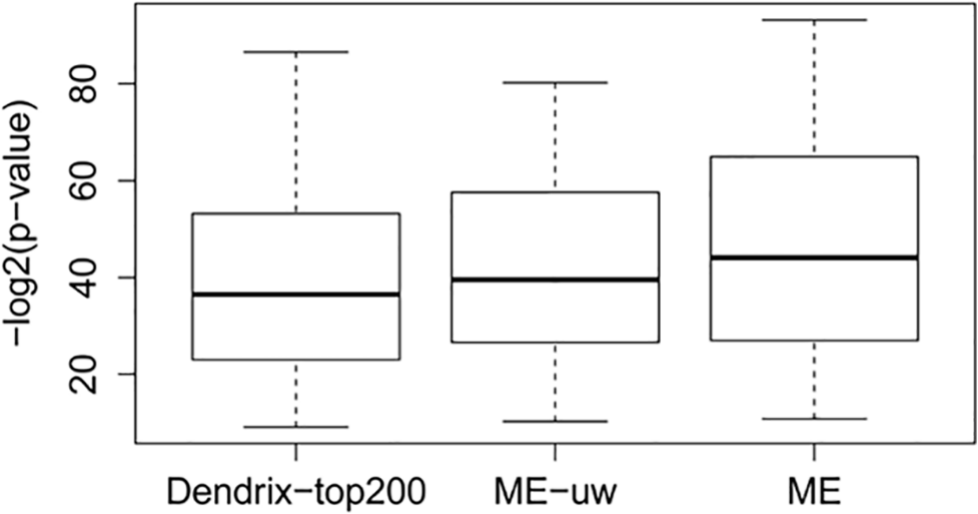
Comparison of heuristic and exact algorithms. Distributions of enrichment *p*-values of the PMs identified by the Dendrix-top200, ME-uw, and ME algorithms.

As shown in Fig 6, the distributions of the enrichment *p*-values of the PMs obtained from the above experiments. As illustrated, the performance of ME-uw is better than that of the Dendrix-top200. Note that the results are in log space, and that therefore a difference of 4.3 in the means corresponds to a 19-fold difference in *p*-values. When we further compared the enrichment *p*-values in a pair-wise manner, we found that the ME-uw outperformed the Dendrix-top200 59 times, whereas the Dendrix-top200 outperformed the ME-uw 23 times. We then compared the performance of ME-uw and Dendrix-top200 using D-weight scores (the objective function explicitly pursued by Dendrix but not by ME). We found that the ME-uw returned a better solution in 33 PMs, whereas the Dendrix-top200 won out in 42 PMs. These results show that, under identical conditions, stochastic algorithms cannot return optimal solutions in a considerable number of cases (here, in at least 33 out of 88 cases). Finally, when the weights of SGAs were considered, the results generated by our ME algorithm further improved upon those of the ME-uw and the Dendrix-top200. We found the mean enrichment *p*-value of our regular ME algorithm to be 40-folds better than that of the Dendrix-top200. Compared with the ME-uw, the ME obtained better PMs for 28 DBSGs, while the ME-uw won out in only 4 cases (with both returning the same PMs in all the remaining cases). By using weight as a parameter, the algorithm can find PMs that are more informative of the pathways regulating an RM in a DBSG.

## Discussion

The challenge of finding the perturbed signaling pathways that underpin the disease mechanisms and heterogeneity of cancers remains one of the most import areas of cancer genomic research. The future of personalized and precision treatment of cancer patients depends on the ability to identify those pathways and to infer their perturbation states in individual patients for the prescribe targeted treatments[3]. While certain auxiliary properties, such as mutual exclusivity and co-expression of candidate members of a pathway, have been applied in searching signaling pathways with a certain degree of success, these properties are not sufficient for the goal of pathway search, as demonstrated in this study. In this study, we proposed a framework that addresses the crux of the pathway-finding problem: identifying proteins that carry a specific *signal*. This is achieved by searching for SGAs (a PM) that carry strong information with respect to the expression state of an RM, which is a surrogate of the state of a signaling pathway. Since genes in a PM are randomly perturbed by nature, a strong association between genes in a pair of PM and RM usually indicates that perturbation of genes in a PM causes differential expressions of genes in the RM. However, it should be noted that such associations may also result from a selection bias, for example, mutation of one gene and over-expression of another are both enriched in a particular tumor subtype, leading to an apparent association even though they do not have a causal relationship. Therefore, further experiment or detailed causal analysis is needed to validate the potential causal relationship between the PM and RM. Our results indicate that the framework is generalizable in that an unbiased program, such as the Dendrix program, can also benefit from the information of the expression state of RMs to find more informative SGA sets exhibiting mutual exclusivity. Therefore, one main contribution of this work is to demonstrate the utility of framework of deconvoluting cellular signals from molecular phenotypic data, e.g., gene expression data, and then enabling pathway-searching programs to simultaneously combine multiple objective functions relevant to pathway search, including signal-oriented objective function proposed here, mutual exclusivity of SGAs, and coexpression of SGA-affected genes.

In this study, we also proposed an exact algorithm to explicitly take into account the amount of information carried by a set of SGAs with respect to an RM by solving the weighted mutually exclusive maximum set cover problem. While it is possible to use the mutual information [63] between the SGA events of a PM and the expression states of a RM—a quantity that is reversely related to the enrichment *p*-value—as the goal in searching for a PM, i.e. searching for a maximum information SGA gene set with respect to a given RM, such formulation will lead to a computational problem that is much more difficult to solve. The current algorithm requires that the members in a PM are mutually exclusive, which is a property taken advantage by our algorithm to yield a practical runtime. In practice, such requirement may be too stringent, leading to omission of some solutions. In our future research, we plan to relax this requirement and allow a small degree of overlap into the solution and design an efficient, exact algorithm to address the new problem.

## Supporting Information

S1 Table Literature search shows that besides AURKA and CCNB1, seven other genes in the RM U_GO0051128 (OV) are related to cancers. (PDF)

S2 Table Genes in the RM U_GO10564 (OV) have been reported to be related to cancers. (PDF)

S3 Table Seventeen RMs from OV and GBM overlap significantly. (PDF)

S4 Table Literature study indicates that most genes in the RMs U_GO0007067 from OV and GBM are related to cancers. (PDF)

S5 Table Kaplan-Meier analysis of RMs from GBM shows that the expression states of 25 RMs are significantly associated with patients’ clinical outcome. (PDF)

S6 Table Using PASTTA (http://trap.molgen.mpg.de/PASTAA.htm), we found that E2F-1 is the most possible transcription factor that regulates the expressions of genes in the RM U_GO0051128 (OV). (PDF)

S7 Table Genes in the PM U_GO0051128 directly interact with well-known oncogenes and tumor suppressors in the table. (PDF)

S1 Fig Kaplan-Meier analysis of top 5 RMs that have strong impact on clinical outcome of TCGA GBM patients. (PDF)

S2 Fig A) Example of genes that are co-amplified with RNF139 and MYC in TCGA OV tumors. B) Expressions of RNF139 in TCGA OV tumors with or without RNF139 amplification. C) Expressions of MYC in TCGA OV tumors with or without MYC amplification.
(PDF)

## References

[1] Hanahan D, Weinberg RA (2000) The hallmarks of cancer. Cell 100: 57–70. 10647931 10.1016/s0092-8674(00)81683-9

[2] Hanahan D, Weinberg RA (2011) Hallmarks of cancer: the next generation. Cell 144: 646–674. 10.1016/j.cell.2011.02.013 21376230

[3] Vogelstein B, Papadopoulos N, Velculescu VE, Zhou S, Diaz LA Jr., et al (2013) Cancer genome landscapes. Science 339: 1546–1558. 10.1126/science.1235122 23539594 PMC3749880

[4] The Cancer Genome Atlas Network (2008) Comprehensive genomic characterization defines human glioblastoma genes and core pathways. Nature 455: 1061–1068. 10.1038/nature07385 18772890 PMC2671642

[5] The Cancer Genome Atlas Network (2011) Integrated genomic analyses of ovarian carcinoma. Nature 474: 609–615. 10.1038/nature10166 21720365 PMC3163504

[6] Ding L, Getz G, Wheeler DA, Mardis ER, McLellan MD, et al (2008) Somatic mutations affect key pathways in lung adenocarcinoma. Nature 455: 1069–1075. 10.1038/nature07423 18948947 PMC2694412

[7] Dees ND, Zhang Q, Kandoth C, Wendl MC, Schierding W, et al (2012) MuSiC: identifying mutational significance in cancer genomes. Genome Res 22: 1589–1598. 10.1101/gr.134635.111 22759861 PMC3409272

[8] Hahn WC, Weinberg RA (2002) Modelling the molecular circuitry of cancer. Nat Rev Cancer 2: 331–341. 12044009 10.1038/nrc795

[9] Basso K, Margolin AA, Stolovitzky G, Klein U, Dalla-Favera R, et al (2005) Reverse engineering of regulatory networks in human B cells. Nat Genet 37: 382–390. 15778709 10.1038/ng1532

[10] Vandin F, Upfal E, Raphael BJ (2011) Algorithms for detecting significantly mutated pathways in cancer. J Comput Biol 18: 507–522. 10.1089/cmb.2010.0265 21385051

[11] Vandin F, Clay P, Upfal E, Raphael BJ (2012) Discovery of mutated subnetworks associated with clinical data in cancer. Pac Symp Biocomput: 55–66. 22174262

[12] Miller CA, Settle SH, Sulman EP, Aldape KD, Milosavljevic A (2011) Discovering functional modules by identifying recurrent and mutually exclusive mutational patterns in tumors. BMC Med Genomics 4: 34 10.1186/1755-8794-4-34 21489305 PMC3102606

[13] Ciriello G, Cerami E, Sander C, Schultz N (2012) Mutual exclusivity analysis identifies oncogenic network modules. Genome Res 22: 398–406. 10.1101/gr.125567.111 21908773 PMC3266046

[14] Zhao J, Zhang S, Wu LY, Zhang XS (2012) Efficient methods for identifying mutated driver pathways in cancer. Bioinformatics 28: 2940–2947. 10.1093/bioinformatics/bts564 22982574

[15] Yamamoto H, Shigematsu H, Nomura M, Lockwood WW, Sato M, et al (2008) PIK3CA mutations and copy number gains in human lung cancers. Cancer Res 68: 6913–6921. 10.1158/0008-5472.CAN-07-5084 18757405 PMC2874836

[16] Vandin F, Upfal E, Raphael BJ (2012) De novo discovery of mutated driver pathways in cancer. Genome Res 22: 375–385. 10.1101/gr.120477.111 21653252 PMC3266044

[17] Leiserson MD, Blokh D, Sharan R, Raphael BJ (2013) Simultaneous identification of multiple driver pathways in cancer. PLoS Comput Biol 9: e1003054 10.1371/journal.pcbi.1003054 23717195 PMC3662702

[18] Ashburner M, Ball CA, Blake JA, Botstein D, Butler H, et al (2000) Gene ontology: tool for the unification of biology. The Gene Ontology Consortium. Nat Genet 25: 25–29. 10802651 10.1038/75556 PMC3037419

[19] Chen V, Lu X (2013) Conceptualization of molecular findings by mining gene annotations. BMC Proceedings Accepted. 10.1186/1753-6561-7-S7-S2 PMC4042834 24564884

[20] Jin B, Lu X (2010) Identifying informative subsets of the Gene Ontology with information bottleneck methods. Bioinformatics 26: 2445–2451. 10.1093/bioinformatics/btq449 20702400 PMC2944202

[21] Lu S, Jin B, Cowart LA, Lu X (2013) From data towards knowledge: revealing the architecture of signaling systems by unifying knowledge mining and data mining of systematic perturbation data. PLoS One 8: e61134 10.1371/journal.pone.0061134 23637789 PMC3634064

[22] Mermel CH, Schumacher SE, Hill B, Meyerson ML, Beroukhim R, et al (2011) GISTIC2.0 facilitates sensitive and confident localization of the targets of focal somatic copy-number alteration in human cancers. Genome Biol 12: R41 10.1186/gb-2011-12-4-r41 21527027 PMC3218867

[23] Ulitsky I, Krishnamurthy A, Karp RM, Shamir R (2010) DEGAS: de novo discovery of dysregulated pathways in human diseases. PLoS One 5: e13367 10.1371/journal.pone.0013367 20976054 PMC2957424

[24] Kim YA, Wuchty S, Przytycka TM (2011) Identifying causal genes and dysregulated pathways in complex diseases. PLoS Comput Biol 7: e1001095 10.1371/journal.pcbi.1001095 21390271 PMC3048384

[25] Kim YA, Salari R, Wuchty S, Przytycka TM (2013) Module cover—a new approach to genotype-phenotype studies. Pac Symp Biocomput: 135–146. PMC3595055 23424119

[26] Karp RM (1972) Reducibility Among Combinatorial Problems: In Complexity of Computer Computations, Miller R. and Thatcher J., Eds., Plenum Press, New York.

[27] Rice JA (2007) Mathematical Statistics and Data Analysis: Duxbury Press.

[28] Lu S, Lu X (2014) An exact algorithm for the weighed mutually exclusive maximum set cover problem. arXiv:14016385. 10.1186/s13015-016-0073-9 PMC4855522 27148394

[29] Downey RG, Fellows MR (1999) Parameterized complexity New York: Springer xv, 533 p. p.

[30] Lu S (2009) Randomized and deterministic parameterized algorithms and their applications in bioinformatics [Ph.D. dissertation]: Texas A&M University.

[31] Gritsko TM, Coppola D, Paciga JE, Yang L, Sun M, et al (2003) Activation and overexpression of centrosome kinase BTAK/Aurora-A in human ovarian cancer. Clin Cancer Res 9: 1420–1426. 12684414

[32] Landen CN Jr., Lin YG, Immaneni A, Deavers MT, Merritt WM, et al (2007) Overexpression of the centrosomal protein Aurora-A kinase is associated with poor prognosis in epithelial ovarian cancer patients. Clin Cancer Res 13: 4098–4104. 17634535 10.1158/1078-0432.CCR-07-0431

[33] Han SS, Tompkins VS, Son DJ, Kamberos NL, Stunz LL, et al (2013) Piperlongumine inhibits LMP1/MYC-dependent mouse B-lymphoma cells. Biochem Biophys Res Commun 436: 660–665. 10.1016/j.bbrc.2013.06.012 23764397 PMC3749779

[34] Rath O, Kozielski F (2012) Kinesins and cancer. Nat Rev Cancer 12: 527–539. 10.1038/nrc3310 22825217

[35] Shubbar E, Kovacs A, Hajizadeh S, Parris TZ, Nemes S, et al (2013) Elevated cyclin B2 expression in invasive breast carcinoma is associated with unfavorable clinical outcome. BMC Cancer 13: 1 10.1186/1471-2407-13-1 23282137 PMC3545739

[36] Fu LJ, Wang B (2013) Investigation of the hub genes and related mechanism in ovarian cancer via bioinformatics analysis. J Ovarian Res 6: 92 10.1186/1757-2215-6-92 24341673 PMC3892009

[37] Curtis C, Shah SP, Chin S-F, Turashvili G, Rueda OM, et al. (2012) The genomic and transcriptomic architecture of 2,000 breast tumours reveals novel subgroups. Nature: 1–7. 10.1038/nature10983 PMC3440846 22522925

[38] Lu S, Cai C, Osmanbeyoglu HU, Chen L, Day R, et al. (2012) Identify Informative Modular Features for Predicting Cancer Clinical Outcomes

[39] Puri S, Bachert C, Fimmel CJ, Linstedt AD (2002) Cycling of early Golgi proteins via the cell surface and endosomes upon lumenal pH disruption. Traffic 3: 641–653. 12191016 10.1034/j.1600-0854.2002.30906.x

[40] Gemmill RM, Bemis LT, Lee JP, Sozen MA, Baron A, et al (2002) The TRC8 hereditary kidney cancer gene suppresses growth and functions with VHL in a common pathway. Oncogene 21: 3507–3516. 12032852 10.1038/sj.onc.1205437

[41] Kosari F, Cheville JC, Ida CM, Karnes RJ, Leontovich AA, et al (2012) Shared gene expression alterations in prostate cancer and histologically benign prostate from patients with prostate cancer. Am J Pathol 181: 34–42. 10.1016/j.ajpath.2012.03.043 22640805 PMC3388167

[42] Chen Y, Hao J, Jiang W, He T, Zhang X, et al (2013) Identifying potential cancer driver genes by genomic data integration. Sci Rep 3: 3538 10.1038/srep03538 24346768 PMC3866686

[43] Beckner M SR, Flowers A, Katira K, D’Souza D, Patil S, Patel R, Nordberg M, Nanda A (2011) Total copy number for 19 amplified genes in atypical/aggressive meningiomas correlates inversely with patient age. The FASEB Journal 25.

[44] Dahlman KB, Parker JS, Shamu T, Hieronymus H, Chapinski C, et al (2012) Modulators of prostate cancer cell proliferation and viability identified by short-hairpin RNA library screening. PLoS One 7: e34414 10.1371/journal.pone.0034414 22509301 PMC3324507

[45] Burke JR, Hura GL, Rubin SM (2012) Structures of inactive retinoblastoma protein reveal multiple mechanisms for cell cycle control. Genes Dev 26: 1156–1166. 10.1101/gad.189837.112 22569856 PMC3371405

[46] Juan G, Cordon-Cardo C (2001) Intranuclear compartmentalization of cyclin E during the cell cycle: disruption of the nucleoplasm-nucleolar shuttling of cyclin E in bladder cancer. Cancer Res 61: 1220–1226. 11221854

[47] Dang CV (2012) MYC on the path to cancer. Cell 149: 22–35. 10.1016/j.cell.2012.03.003 22464321 PMC3345192

[48] Chu IM, Hengst L, Slingerland JM (2008) The Cdk inhibitor p27 in human cancer: prognostic potential and relevance to anticancer therapy. Nat Rev Cancer 8: 253–267. 10.1038/nrc2347 18354415

[49] Kaelin WG Jr. (2002) Molecular basis of the VHL hereditary cancer syndrome. Nat Rev Cancer 2: 673–682. 12209156 10.1038/nrc885

[50] Zack TI, Schumacher SE, Carter SL, Cherniack AD, Saksena G, et al (2013) Pan-cancer patterns of somatic copy number alteration. Nat Genet 45: 1134–1140. 10.1038/ng.2760 24071852 PMC3966983

[51] Zhao Y, Yu H, Hu W (2014) The regulation of MDM2 oncogene and its impact on human cancers. Acta Biochim Biophys Sin (Shanghai) 46: 180–189. 10.1093/abbs/gmt147 24389645 PMC3932834

[52] Wang H, Zeng X, Oliver P, Le LP, Chen J, et al (1999) MDM2 oncogene as a target for cancer therapy: An antisense approach. Int J Oncol 15: 653–660. 10493945

[53] Wade M, Li YC, Wahl GM (2013) MDM2, MDMX and p53 in oncogenesis and cancer therapy. Nat Rev Cancer 13: 83–96. 10.1038/nrc3430 23303139 PMC4161369

[54] Ray RM, Bhattacharya S, Johnson LR (2011) Mdm2 inhibition induces apoptosis in p53 deficient human colon cancer cells by activating p73- and E2F1-mediated expression of PUMA and Siva-1. Apoptosis 16: 35–44. 10.1007/s10495-010-0538-0 20812030

[55] Kojima K, Konopleva M, Samudio IJ, Shikami M, Cabreira-Hansen M, et al (2005) MDM2 antagonists induce p53-dependent apoptosis in AML: implications for leukemia therapy. Blood 106: 3150–3159. 16014563 10.1182/blood-2005-02-0553 PMC1895324

[56] Costa B, Bendinelli S, Gabelloni P, Da Pozzo E, Daniele S, et al (2013) Human glioblastoma multiforme: p53 reactivation by a novel MDM2 inhibitor. PLoS One 8: e72281 10.1371/journal.pone.0072281 23977270 PMC3747081

[57] Listernick R, Charrow J, Gutmann DH (1999) Intracranial gliomas in neurofibromatosis type 1. Am J Med Genet 89: 38–44. 10469435 10.1002/(sici)1096-8628(19990326)89:1<38::aid-ajmg8>3.0.co;2-m

[58] Parrinello S, Lloyd AC (2009) Neurofibroma development in NF1—insights into tumour initiation. Trends Cell Biol 19: 395–403. 10.1016/j.tcb.2009.05.003 19615906

[59] Shapira S, Barkan B, Friedman E, Kloog Y, Stein R (2007) The tumor suppressor neurofibromin confers sensitivity to apoptosis by Ras-dependent and Ras-independent pathways. Cell Death Differ 14: 895–906. 17096025 10.1038/sj.cdd.4402057

[60] Pina C, May G, Soneji S, Hong D, Enver T (2008) MLLT3 regulates early human erythroid and megakaryocytic cell fate. Cell Stem Cell 2: 264–273. 10.1016/j.stem.2008.01.013 18371451

[61] Fuentealba RA, Liu Q, Kanekiyo T, Zhang J, Bu G (2009) Low density lipoprotein receptor-related protein 1 promotes anti-apoptotic signaling in neurons by activating Akt survival pathway. J Biol Chem 284: 34045–34053. 10.1074/jbc.M109.021030 19815552 PMC2797175

[62] Yahiro K, Satoh M, Nakano M, Hisatsune J, Isomoto H, et al (2012) Low-density lipoprotein receptor-related protein-1 (LRP1) mediates autophagy and apoptosis caused by Helicobacter pylori VacA. J Biol Chem 287: 31104–31115. 10.1074/jbc.M112.387498 22822085 PMC3438942

[63] Cover TM, Thomas JA (2006) Elements of Information Theory: Wiley-Interscience.

