# Supplementary material for: Identifying Driver Genomic Alterations in Cancers by Searching Minimum-Weight, Mutually Exclusive Sets
Source: PLoS Comput Biol. 2015 Aug 28;11(8):e1004257. doi: 10.1371/journal.pcbi.1004257 (PMC4552843; doi:10.1371/journal.pcbi.1004257)
Supplement: S1 Table — (PDF) [file pcbi.1004257.s001.pdf]

Table S1- Literature search shows that besides AURKA and CCNB1, seven other genes in the RM U_GO0051128 (OV) are related to cancers.

Gene	Reference
CHEK1	[1-3]
EPHB3	[4,5]
NEK2	[6,7]
STMN1	[8-11]
TACC3	[12]
THBS2	[13]
TWIST1	[14-16]

References for the Supplementary

1. Desmarais JA, Hoffmann MJ, Bingham G, Gagou ME, Meuth M, et al. (2012) Human embryonic stem cells fail to activate CHK1 and commit to apoptosis in response to DNA replication stress. Stem Cells 30: 1385-1393.
2. Herman-Antosiewicz A, Singh SV (2005) Checkpoint kinase 1 regulates diallyl trisulfide-induced mitotic arrest in human prostate cancer cells. J Biol Chem 280: 28519-28528.
3. Connell CM, Shibata A, Tookman LA, Archibald KM, Flak MB, et al. (2011) Genomic DNA damage and ATR-Chk1 signaling determine oncolytic adenoviral efficacy in human ovarian cancer cells. J Clin Invest 121: 1283-1297.
4. Ji XD, Li G, Feng YX, Zhao JS, Li JJ, et al. (2011) EphB3 is overexpressed in non-small-cell lung cancer and promotes tumor metastasis by enhancing cell survival and migration. Cancer Res 71: 1156-1166.
5. Chiu ST, Chang KJ, Ting CH, Shen HC, Li H, et al. (2009) Over-expression of EphB3 enhances cell-cell contacts and suppresses tumor growth in HT-29 human colon cancer cells. Carcinogenesis 30: 1475-1486.
6. Liu Z, Wang Y, Wang S, Zhang J, Zhang F, et al. (2012) Nek2C functions as a tumor promoter in human breast tumorigenesis. Int J Mol Med 30: 775-782.
7. Tsunoda N, Kokuryo T, Oda K, Senga T, Yokoyama Y, et al. (2009) Nek2 as a novel molecular target for the treatment of breast carcinoma. Cancer Sci 100: 111-116.
8. Trovik J, Wik E, Stefansson IM, Marcickiewicz J, Tingulstad S, et al. (2011) Stathmin overexpression identifies high-risk patients and lymph node metastasis in endometrial cancer. Clin Cancer Res 17: 3368-3377.
9. Jeon TY, Han ME, Lee YW, Lee YS, Kim GH, et al. (2010) Overexpression of stathmin1 in the diffuse type of gastric cancer and its roles in proliferation and migration of gastric cancer cells. Br J Cancer 102: 710-718.
10. Sonego M, Schiappacassi M, Lovisa S, Dall'Acqua A, Bagnoli M, et al. (2013) Stathmin regulates mutant p53 stability and transcriptional activity in ovarian cancer. EMBO Mol Med 5: 707-722.
11. Wei SH, Lin F, Wang X, Gao P, Zhang HZ (2008) Prognostic significance of stathmin expression in correlation with metastasis and clinicopathological characteristics in human ovarian carcinoma. Acta Histochem 110: 59-65.

12. Yim EK, Tong SY, Ho EM, Bae JH, Um SJ, et al. (2009) Anticancer effects on TACC3 by treatment of paclitaxel in HPV-18 positive cervical carcinoma cells. Oncol Rep 21: 549-557.
13. Linton A, Cheng YY, Griggs K, Kirschner MB, Gattani S, et al. (2014) An RNAi-based screen reveals PLK1, CDK1 and NDC80 as potential therapeutic targets in malignant pleural mesothelioma. Br J Cancer 110: 510-519.
14. Wang WS, Yang XS, Xia M, Jiang HY, Hou JQ (2012) Silencing of twist expression by RNA interference suppresses epithelial-mesenchymal transition, invasion, and metastasis of ovarian cancer. Asian Pac J Cancer Prev 13: 4435-4439.
15. Yin G, Alvero AB, Craveiro V, Holmberg JC, Fu HH, et al. (2013) Constitutive proteasomal degradation of TWIST-1 in epithelial-ovarian cancer stem cells impacts differentiation and metastatic potential. Oncogene 32: 39-49.
16. Sun T, Zhao N, Zhao XL, Gu Q, Zhang SW, et al. (2010) Expression and functional significance of Twist1 in hepatocellular carcinoma: its role in vasculogenic mimicry. Hepatology 51: 545-556.
