# Supplementary material for: Identifying Driver Genomic Alterations in Cancers by Searching Minimum-Weight, Mutually Exclusive Sets
Source: PLoS Comput Biol. 2015 Aug 28;11(8):e1004257. doi: 10.1371/journal.pcbi.1004257 (PMC4552843; doi:10.1371/journal.pcbi.1004257)
Supplement: S2 Table — (PDF) [file pcbi.1004257.s002.pdf]

Table S2 - Genes in the RM U_GO10564 (OV) have been reported to be related to cancers

Gene	Reference
BIRC5	[1,2]
CCNB2	[3-5]
CDC7	[6-8]
CENPE	[9]
CENPF	[10]
CHEK1	[11-13]
NEK2	[14,15]
TIMELESS	[16,17]
UBE2C	[18-20]

References for the Supplementary

1. Cascione L, Gasparini P, Lovat F, Carasi S, Pulvirenti A, et al. (2013) Integrated microRNA and mRNA signatures associated with survival in triple negative breast cancer. PLoS One 8: e55910.
2. Boidot R, Vegran F, Jacob D, Chevrier S, Gangneux N, et al. (2008) The expression of BIRC5 is correlated with loss of specific chromosomal regions in breast carcinomas. Genes Chromosomes Cancer 47: 299-308.
3. Shubbar E, Kovacs A, Hajizadeh S, Parris TZ, Nemes S, et al. (2013) Elevated cyclin B2 expression in invasive breast carcinoma is associated with unfavorable clinical outcome. BMC Cancer 13: 1.
4. Stav D, Bar I, Sandbank J (2007) Usefulness of CDK5RAP3, CCNB2, and RAGE genes for the diagnosis of lung adenocarcinoma. Int J Biol Markers 22: 108-113.
5. Mo ML, Chen Z, Li J, Li HL, Sheng Q, et al. (2010) Use of serum circulating CCNB2 in cancer surveillance. Int J Biol Markers 25: 236-242.
6. Cheng AN, Jiang SS, Fan CC, Lo YK, Kuo CY, et al. (2013) Increased Cdc7 expression is a marker of oral squamous cell carcinoma and overexpression of Cdc7 contributes to the resistance to DNA-damaging agents. Cancer Lett 337: 218-225.
7. Kulkarni AA, Kingsbury SR, Tudzarova S, Hong HK, Loddo M, et al. (2009) Cdc7 kinase is a predictor of survival and a novel therapeutic target in epithelial ovarian carcinoma. Clin Cancer Res 15: 2417-2425.
8. Bonte D, Lindvall C, Liu H, Dykema K, Furge K, et al. (2008) Cdc7-Dbf4 kinase overexpression in multiple cancers and tumor cell lines is correlated with p53 inactivation. Neoplasia 10: 920-931.
9. Wood KW, Chua P, Sutton D, Jackson JR (2008) Centromere-associated protein E: a motor that puts the brakes on the mitotic checkpoint. Clin Cancer Res 14: 7588-7592.
10. Cao JY, Liu L, Chen SP, Zhang X, Mi YJ, et al. (2010) Prognostic significance and therapeutic implications of centromere protein F expression in human nasopharyngeal carcinoma. Mol Cancer 9: 237.
11. Herman-Antosiewicz A, Singh SV (2005) Checkpoint kinase 1 regulates diallyl trisulfide-induced mitotic arrest in human prostate cancer cells. J Biol Chem 280: 28519-28528.

12. Desmarais JA, Hoffmann MJ, Bingham G, Gagou ME, Meuth M, et al. (2012) Human embryonic stem cells fail to activate CHK1 and commit to apoptosis in response to DNA replication stress. Stem Cells 30: 1385-1393.
13. Connell CM, Shibata A, Tookman LA, Archibald KM, Flak MB, et al. (2011) Genomic DNA damage and ATR-Chk1 signaling determine oncolytic adenoviral efficacy in human ovarian cancer cells. J Clin Invest 121: 1283-1297.
14. Liu Z, Wang Y, Wang S, Zhang J, Zhang F, et al. (2012) Nek2C functions as a tumor promoter in human breast tumorigenesis. Int J Mol Med 30: 775-782.
15. Wang S, Li W, Liu N, Zhang F, Liu H, et al. (2012) Nek2A contributes to tumorigenic growth and possibly functions as potential therapeutic target for human breast cancer. J Cell Biochem 113: 1904-1914.
16. Yoshida K, Sato M, Hase T, Elshazley M, Yamashita R, et al. (2013) TIMELESS is overexpressed in lung cancer and its expression correlates with poor patient survival. Cancer Sci 104: 171-177.
17. Fu A, Leaderer D, Zheng T, Hoffman AE, Stevens RG, et al. (2012) Genetic and epigenetic associations of circadian gene TIMELESS and breast cancer risk. Mol Carcinog 51: 923-929.
18. Bose MV, Gopisetty G, Selvaluxmy G, Rajkumar T (2012) Dominant negative Ubiquitin-conjugating enzyme E2C sensitizes cervical cancer cells to radiation. Int J Radiat Biol 88: 629-634.
19. Psyrri A, Kalogeras KT, Kronenwett R, Wirtz RM, Batistatou A, et al. (2012) Prognostic significance of UBE2C mRNA expression in high-risk early breast cancer. A Hellenic Cooperative Oncology Group (HeCOG) Study. Ann Oncol 23: 1422-1427.
20. Morikawa T, Kawai T, Abe H, Kume H, Homma Y, et al. (2013) UBE2C is a marker of unfavorable prognosis in bladder cancer after radical cystectomy. Int J Clin Exp Pathol 6: 1367-1374.
