# Supplementary material for: Identifying Driver Genomic Alterations in Cancers by Searching Minimum-Weight, Mutually Exclusive Sets
Source: PLoS Comput Biol. 2015 Aug 28;11(8):e1004257. doi: 10.1371/journal.pcbi.1004257 (PMC4552843; doi:10.1371/journal.pcbi.1004257)
Supplement: S3 Table — (PDF) [file pcbi.1004257.s003.pdf]

Table S3 - Seventeen RMs from OV and GBM overlap significantly

RM_ID	GO term gene count	RM size (OV)	RM size (GBM)	Common count	Overlap p-value	q_values
U_GO0022403	505	31	35	22	1.42E-22	5.40E-21
U_GO0007067	186	17	18	15	2.27E-17	4.31E-16
U_GO0051726	527	22	27	15	6.04E-17	7.65E-16
U_GO0000278	319	22	30	17	3.83E-16	3.64E-15
U_GO0006259	619	20	19	11	9.71E-14	6.84E-13
U_GO0000226	180	15	15	12	1.19E-13	6.84E-13
U_GO0006974	554	14	19	10	1.26E-13	6.84E-13
U_GO0000087	94	15	19	14	9.42E-12	4.47E-11
U_GO0000075	216	18	19	12	4.88E-11	2.06E-10
U_GO0000236	84	14	18	13	1.79E-10	6.74E-10
U_GO0006996	1116	13	19	7	1.95E-10	6.74E-10
U_GO0034984	490	12	19	8	4.43E-10	1.40E-09
U_GO0009615	194	13	17	9	1.67E-08	4.88E-08
U_GO0009611	467	10	30	6	7.45E-06	2.02E-05
U_GO0030198	122	11	19	8	9.88E-06	2.50E-05
U_GO0007242	1286	14	18	4	2.47E-05	5.87E-05
U_GO0007596	460	10	21	5	2.63E-05	5.88E-05
