# Supplementary material for: Identifying Driver Genomic Alterations in Cancers by Searching Minimum-Weight, Mutually Exclusive Sets
Source: PLoS Comput Biol. 2015 Aug 28;11(8):e1004257. doi: 10.1371/journal.pcbi.1004257 (PMC4552843; doi:10.1371/journal.pcbi.1004257)
Supplement: S4 Table — (PDF) [file pcbi.1004257.s004.pdf]

Table S4 - Literature study indicates that most genes in the RMs U_GO0007067 from OV and GBM are related to cancers

Gene	Reference		Gene	Reference
ASPM	[1,2]		OIP5	[3,4]
AURKA	[5-7]		PBK	[8]
BIRC5	[9,10]		PTTG1	[11,12]
BUB1B	[13,14]		TIMELESS	[15,16]
CCNA2	[17,18]		TPX2	[7,19]
CCNB2	[20,21]		UBE2C	[22-24]
CEP55	[25]		WEE1	[26,27]
KIF23	[28]			
KIF2C	[29,30]			
NDC80	[31,32]			

References for the Supplementary

1. Wang WY, Hsu CC, Wang TY, Li CR, Hou YC, et al. (2013) A gene expression signature of epithelial tubulogenesis and a role for ASPM in pancreatic tumor progression. Gastroenterology 145: 1110-1120.
2. Vulcani-Freitas TM, Saba-Silva N, Cappellano A, Cavalheiro S, Marie SK, et al. (2011) ASPM gene expression in medulloblastoma. Childs Nerv Syst 27: 71-74.
3. Chun HK, Chung KS, Kim HC, Kang JE, Kang MA, et al. (2010) OIP5 is a highly expressed potential therapeutic target for colorectal and gastric cancers. BMB Rep 43: 349-354.
4. Koinuma J, Akiyama H, Fujita M, Hosokawa M, Tsuchiya E, et al. (2012) Characterization of an Opa interacting protein 5 involved in lung and esophageal carcinogenesis. Cancer Sci 103: 577-586.
5. Do TV, Xiao F, Bickel LE, Klein-Szanto AJ, Pathak HB, et al. (2014) Aurora kinase A mediates epithelial ovarian cancer cell migration and adhesion. Oncogene 33: 539-549.
6. Goos JA, Coupe VM, Diosdado B, Delis-Van Diemen PM, Karga C, et al. (2013) Aurora kinase A (AURKA) expression in colorectal cancer liver metastasis is associated with poor prognosis. Br J Cancer 109: 2445-2452.
7. Sillars-Hardebol AH, Carvalho B, Tijssen M, Belien JA, de Wit M, et al. (2012) TPX2 and AURKA promote 20q amplicon-driven colorectal adenoma to carcinoma progression. Gut 61: 1568-1575.
8. Simons-Evelyn M, Bailey-Dell K, Toretsky JA, Ross DD, Fenton R, et al. (2001) PBK/TOPK is a novel mitotic kinase which is upregulated in Burkitt's lymphoma and other highly proliferative malignant cells. Blood Cells Mol Dis 27: 825-829.
9. Cascione L, Gasparini P, Lovat F, Carasi S, Pulvirenti A, et al. (2013) Integrated microRNA and mRNA signatures associated with survival in triple negative breast cancer. PLoS One 8: e55910.
10. Boidot R, Vegran F, Jacob D, Chevrier S, Gangneux N, et al. (2008) The expression of BIRC5 is correlated with loss of specific chromosomal regions in breast carcinomas. Genes Chromosomes Cancer 47: 299-308.

11. Demeure MJ, Coan KE, Grant CS, Komorowski RA, Stephan E, et al. (2013) PTTG1 overexpression in adrenocortical cancer is associated with poor survival and represents a potential therapeutic target. Surgery 154: 1405-1416; discussion 1416.
12. Salehi F, Scheithauer BW, Sharma S, Kovacs K, Lloyd RV, et al. (2013) Immunohistochemical expression of PTTG in brain tumors. Anticancer Res 33: 119-122.
13. Ding Y, Hubert CG, Herman J, Corrin P, Toledo CM, et al. (2013) Cancer-Specific requirement for BUB1B/BUBR1 in human brain tumor isolates and genetically transformed cells. Cancer Discov 3: 198-211.
14. Ando K, Kakeji Y, Kitao H, Iimori M, Zhao Y, et al. (2010) High expression of BUBR1 is one of the factors for inducing DNA aneuploidy and progression in gastric cancer. Cancer Sci 101: 639-645.
15. Yoshida K, Sato M, Hase T, Elshazley M, Yamashita R, et al. (2013) TIMELESS is overexpressed in lung cancer and its expression correlates with poor patient survival. Cancer Sci 104: 171-177.
16. Fu A, Leaderer D, Zheng T, Hoffman AE, Stevens RG, et al. (2012) Genetic and epigenetic associations of circadian gene TIMELESS and breast cancer risk. Mol Carcinog 51: 923-929.
17. Santala S, Talvensaari-Mattila A, Soini Y, Honkavuori-Toivola M, Santala M (2014) High expression of cyclin A is associated with poor prognosis in endometrial endometrioid adenocarcinoma. Tumour Biol 35: 5395-5399.
18. Dunican DS, McWilliam P, Tighe O, Parle-McDermott A, Croke DT (2002) Gene expression differences between the microsatellite instability (MIN) and chromosomal instability (CIN) phenotypes in colorectal cancer revealed by high-density cDNA array hybridization. Oncogene 21: 3253-3257.
19. Li B, Qi XQ, Chen X, Huang X, Liu GY, et al. (2010) Expression of targeting protein for Xenopus kinesin-like protein 2 is associated with progression of human malignant astrocytoma. Brain Res 1352: 200-207.
20. Stav D, Bar I, Sandbank J (2007) Usefulness of CDK5RAP3, CCNB2, and RAGE genes for the diagnosis of lung adenocarcinoma. Int J Biol Markers 22: 108-113.
21. Shubbar E, Kovacs A, Hajizadeh S, Parris TZ, Nemes S, et al. (2013) Elevated cyclin B2 expression in invasive breast carcinoma is associated with unfavorable clinical outcome. BMC Cancer 13: 1.
22. Morikawa T, Kawai T, Abe H, Kume H, Homma Y, et al. (2013) UBE2C is a marker of unfavorable prognosis in bladder cancer after radical cystectomy. Int J Clin Exp Pathol 6: 1367-1374.
23. Psyrri A, Kalogeras KT, Kronenwett R, Wirtz RM, Batistatou A, et al. (2012) Prognostic significance of UBE2C mRNA expression in high-risk early breast cancer. A Hellenic Cooperative Oncology Group (HeCOG) Study. Ann Oncol 23: 1422-1427.
24. Bose MV, Gopisetty G, Selvaluxmy G, Rajkumar T (2012) Dominant negative Ubiquitin-conjugating enzyme E2C sensitizes cervical cancer cells to radiation. Int J Radiat Biol 88: 629-634.
25. Waseem A, Ali M, Odell EW, Fortune F, Teh MT (2010) Downstream targets of FOXM1: CEP55 and HELLS are cancer progression markers of head and neck squamous cell carcinoma. Oral Oncol 46: 536-542.
26. Mir SE, De Witt Hamer PC, Krawczyk PM, Balaj L, Claes A, et al. (2010) In silico analysis of kinase expression identifies WEE1 as a gatekeeper against mitotic catastrophe in glioblastoma. Cancer Cell 18: 244-257.
27. Magnussen GI, Holm R, Emilsen E, Rosnes AK, Slipicevic A, et al. (2012) High expression of Wee1 is associated with poor disease-free survival in malignant melanoma: potential for targeted therapy. PLoS One 7: e38254.
28. Takahashi S, Fusaki N, Ohta S, Iwahori Y, Iizuka Y, et al. (2012) Downregulation of KIF23 suppresses glioma proliferation. J Neurooncol 106: 519-529.

29. Gnjatic S, Cao Y, Reichelt U, Yekebas EF, Nolker C, et al. (2010) NY-CO-58/KIF2C is overexpressed in a variety of solid tumors and induces frequent T cell responses in patients with colorectal cancer. Int J Cancer 127: 381-393.
30. Shimo A, Tanikawa C, Nishidate T, Lin ML, Matsuda K, et al. (2008) Involvement of kinesin family member 2C/mitotic centromere-associated kinesin overexpression in mammary carcinogenesis. Cancer Sci 99: 62-70.
31. Bieche I, Vacher S, Lallemand F, Tozlu-Kara S, Bennani H, et al. (2011) Expression analysis of mitotic spindle checkpoint genes in breast carcinoma: role of NDC80/HEC1 in early breast tumorigenicity, and a two-gene signature for aneuploidy. Mol Cancer 10: 23.
32. Linton A, Cheng YY, Griggs K, Kirschner MB, Gattani S, et al. (2014) An RNAi-based screen reveals PLK1, CDK1 and NDC80 as potential therapeutic targets in malignant pleural mesothelioma. Br J Cancer 110: 510-519.
