# Supplementary material for: Identifying Driver Genomic Alterations in Cancers by Searching Minimum-Weight, Mutually Exclusive Sets
Source: PLoS Comput Biol. 2015 Aug 28;11(8):e1004257. doi: 10.1371/journal.pcbi.1004257 (PMC4552843; doi:10.1371/journal.pcbi.1004257)
Supplement: S5 Table — (PDF) [file pcbi.1004257.s005.pdf]

Table S5 - Kaplan-Meier analysis of RMs from GBM shows that the expression states of 25 RMs are significantly associated with patients' clinical outcome

RM	p-value	q-value		RM	p-value	q-value
U_GO0050793	3.01E-05	0.0020197		U_GO0010646	0.004761394	0.022821
U_GO0042592	6.87E-05	0.0023049		U_GO0009966	0.005467702	0.024459
U_GO0048513	0.0001818	0.0034745		U_GO0012501	0.006062865	0.025426
U_GO0009611	0.00020712	0.0034745		U_GO0006915	0.007285621	0.026724
U_GO0007165	0.00028114	0.0037728		U_GO0048545	0.007316108	0.026724
U_GO0043067	0.00096769	0.0108221		U_GO0048878	0.007849759	0.026724
U_GO0009725	0.00118812	0.011389		U_GO0043062	0.007965369	0.026724
U_GO0006954	0.00178206	0.0149471		U_GO0030198	0.008670935	0.027276
D_GO0043067	0.00242886	0.0181085		U_GO0032496	0.008943006	0.027276
U_GO0022603	0.00298315	0.020017		U_GO0051270	0.01166546	0.033899
U_GO0051239	0.00371628	0.021917		U_GO0007596	0.01212466	0.033899
D_GO0007166	0.00391957	0.021917		U_GO0043066	0.01666711	0.044735
U_GO0007242	0.0043813	0.0226143				
