# Supplementary material for: Identifying Driver Genomic Alterations in Cancers by Searching Minimum-Weight, Mutually Exclusive Sets
Source: PLoS Comput Biol. 2015 Aug 28;11(8):e1004257. doi: 10.1371/journal.pcbi.1004257 (PMC4552843; doi:10.1371/journal.pcbi.1004257)
Supplement: S6 Table — (PDF) [file pcbi.1004257.s006.pdf]

Table S6 - Using PASTTA (http://trap.molgen.mpg.de/PASTAA.htm), we found that E2F-1 is the most possible transcription factor that regulates the expressions of genes in the RM U_GO0051128 (OV)

Rank	Matrix	Transcription Factor	Association Score	P-Value
1	E2F_Q6_01	Dp-1 , E2f-1	3.945	6.87E-04
2	CACBINDINGPROTEIN_Q6	N/A	3.711	1.12E-03
3	E2F_Q3_01	Dp-1 , E2f-1	3.711	1.12E-03
4	E2F1_Q4_01	Dp-1 , E2f-1	3.514	1.65E-03
5	E2F1_Q4	E2f-1	3.475	1.76E-03
6	AP2ALPHA_03	N/A	3.366	2.16E-03
7	E2F_Q4_01	Dp-1 , E2f-1	3.344	2.21E-03
8	MYCMAX_02	Max1 , C-myc	2.822	7.32E-03
9	USF_Q6_01	Usf-1 , Usf1	2.814	7.38E-03
10	PU1_Q6	Pu.1	2.657	1.08E-02
11	PAX4_03	Pax-4a	2.587	1.15E-02
12	E2F_03	E2f-1	2.575	1.18E-02
