# Supplementary material for: Identifying Driver Genomic Alterations in Cancers by Searching Minimum-Weight, Mutually Exclusive Sets
Source: PLoS Comput Biol. 2015 Aug 28;11(8):e1004257. doi: 10.1371/journal.pcbi.1004257 (PMC4552843; doi:10.1371/journal.pcbi.1004257)
Supplement: S7 Table — (PDF) [file pcbi.1004257.s007.pdf]

Table S7 - Genes in the PM U_GO0051128 directly interact with well-known oncogenes in the table

Gene	Reference
TP53	[1,2]
MDM4	[3,4]
CCND1	[5,6]
CCND2	[7,8]
MYC	[9,10]
E2F3	[11]
E2F5	[12,13]
BRCA2	[14-17]
PTEN	[18,19]
MET	[20-22]
COPS5	[23]

References for the Supplementary

1. Jesionek-Kupnicka D, Szybka M, Malachowska B, Fendler W, Potemski P, et al. (2014) TP53 promoter methylation in primary glioblastoma: relationship with TP53 mRNA and protein expression and mutation status. DNA Cell Biol 33: 217-226.
2. Zhang GN, Liu H, Huang JM, Wang L, Zhao JS, et al. (2014) TP53 K351N mutation-associated platinum resistance after neoadjuvant chemotherapy in patients with advanced ovarian cancer. Gynecol Oncol 132: 752-757.
3. Yu H, Sturgis EM, Liu Z, Wang LE, Wei Q, et al. (2012) Modifying effect of MDM4 variants on risk of HPV16-associated squamous cell carcinoma of oropharynx. Cancer 118: 1684-1692.
4. Yu H, Wang LE, Liu Z, Wei S, Li G, et al. (2011) Polymorphisms of MDM4 and risk of squamous cell carcinoma of the head and neck. Pharmacogenet Genomics 21: 388-396.
5. Dai J, Zhang PH, Liu PS, Qu HH (2012) [Expressions and significance of cyclinD1 in epithelial ovarian cancer cell 3AO]. Zhonghua Yi Xue Za Zhi 92: 351-353.
6. Zeybek U, Yaylim I, Ozkan NE, Korkmaz G, Turan S, et al. (2013) Cyclin D1 gene G870A variants and primary brain tumors. Asian Pac J Cancer Prev 14: 4101-4106.
7. Goode EL, Fridley BL, Vierkant RA, Cunningham JM, Phelan CM, et al. (2009) Candidate gene analysis using imputed genotypes: cell cycle single-nucleotide polymorphisms and ovarian cancer risk. Cancer Epidemiol Biomarkers Prev 18: 935-944.
8. Koyama-Nasu R, Nasu-Nishimura Y, Todo T, Ino Y, Saito N, et al. (2013) The critical role of cyclin D2 in cell cycle progression and tumorigenicity of glioblastoma stem cells. Oncogene 32: 3840-3845.
9. Terunuma A, Putluri N, Mishra P, Mathe EA, Dorsey TH, et al. (2014) MYC-driven accumulation of 2-hydroxyglutarate is associated with breast cancer prognosis. J Clin Invest 124: 398-412.
10. Civenni G, Malek A, Albino D, Garcia-Escudero R, Napoli S, et al. (2013) RNAi-mediated silencing of Myc transcription inhibits stem-like cell maintenance and tumorigenicity in prostate cancer. Cancer Res 73: 6816-6827.

11. Reimer D, Hubalek M, Riedle S, Skvortsov S, Erdel M, et al. (2010) E2F3a is critically involved in epidermal growth factor receptor-directed proliferation in ovarian cancer. Cancer Res 70: 4613-4623.
12. Kothandaraman N, Bajic VB, Brendan PN, Huak CY, Keow PB, et al. (2010) E2F5 status significantly improves malignancy diagnosis of epithelial ovarian cancer. BMC Cancer 10: 64.
13. Ishimoto T, Shiozaki A, Ichikawa D, Fujiwara H, Konishi H, et al. (2013) E2F5 as an independent prognostic factor in esophageal squamous cell carcinoma. Anticancer Res 33: 5415-5420.
14. Darehdori AS, Dastjerdi MN, Dahim H, Slahshoor M, Babazadeh Z, et al. (2012) Lack of significance of the BRCA2 promoter methylation status in different genotypes of the MTHFR a1298c polymorphism in ovarian cancer cases in Iran. Asian Pac J Cancer Prev 13: 1833-1836.
15. Root K, Armaghany T (2012) Solitary brain metastasis in a patient with ovarian cancer with BRCA2 mutation. J Clin Oncol 30: e239-240.
16. Ruiz de Garibay G, Gutierrez-Enriquez S, Garre P, Bonache S, Romero A, et al. (2012) Characterization of four novel BRCA2 large genomic rearrangements in Spanish breast/ovarian cancer families: review of the literature, and reevaluation of the genetic mechanisms involved in their origin. Breast Cancer Res Treat 133: 273-283.
17. Balabas A, Skasko E, Nowakowska D, Niwinska A, Blecharz P (2010) Novel germline mutations in BRCA2 gene among breast and breast-ovarian cancer families from Poland. Fam Cancer 9: 267-274.
18. Dean SJ, Perks CM, Holly JM, Bhoo-Pathy N, Looi LM, et al. (2014) Loss of PTEN expression is associated with IGFBP2 expression, younger age, and late stage in triple-negative breast cancer. Am J Clin Pathol 141: 323-333.
19. Mester JL, Moore RA, Eng C (2013) PTEN germline mutations in patients initially tested for other hereditary cancer syndromes: would use of risk assessment tools reduce genetic testing? Oncologist 18: 1083-1090.
20. Yamashita Y, Akatsuka S, Shinjo K, Yatabe Y, Kobayashi H, et al. (2013) Met is the most frequently amplified gene in endometriosis-associated ovarian clear cell adenocarcinoma and correlates with worsened prognosis. PLoS One 8: e57724.
21. Marchion DC, Bicaku E, Xiong Y, Bou Zgheib N, Al Sawah E, et al. (2013) A novel c-Met inhibitor, MK8033, synergizes with carboplatin plus paclitaxel to inhibit ovarian cancer cell growth. Oncol Rep 29: 2011-2018.
22. Yamamoto S, Tsuda H, Miyai K, Takano M, Tamai S, et al. (2012) Accumulative copy number increase of MET drives tumor development and histological progression in a subset of ovarian clear-cell adenocarcinomas. Mod Pathol 25: 122-130.
23. Adler AS, Littlepage LE, Lin M, Kawahara TL, Wong DJ, et al. (2008) CSN5 isopeptidase activity links COP9 signalosome activation to breast cancer progression. Cancer Res 68: 506-515.
