# Supplementary material for: Identifying Driver Genomic Alterations in Cancers by Searching Minimum-Weight, Mutually Exclusive Sets
Source: PLoS Comput Biol. 2015 Aug 28;11(8):e1004257. doi: 10.1371/journal.pcbi.1004257 (PMC4552843; doi:10.1371/journal.pcbi.1004257)
Supplement: S1 Fig — (PDF) [file pcbi.1004257.s008.pdf]

Figure S1 – Kaplan-Meier analysis of top 5 RMs that have strong impact on clinical outcome of TCGA GBM patients

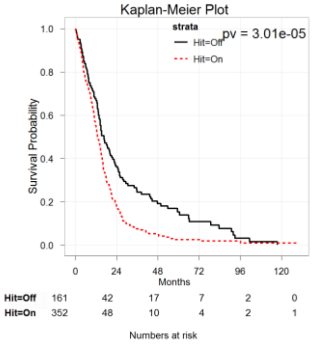

RM U_GO0050793: regulation of developmental process

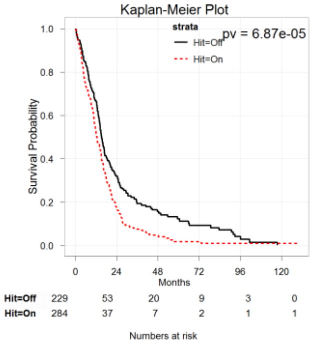

RM U_GO0042592: homeostatic process

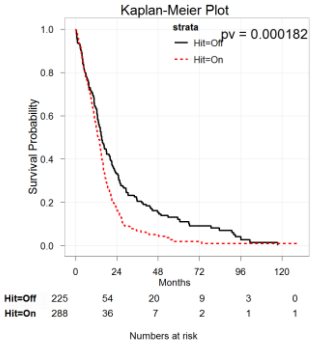

RM U_GO0048513: organ development

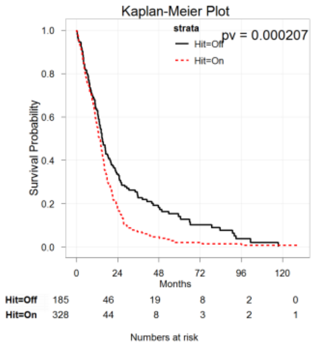

RM U_GO0009611: response to wounding

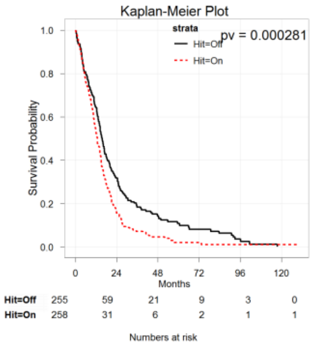

RM U_GO0007165: signal transduction
