# Supplementary material for: Identifying Driver Genomic Alterations in Cancers by Searching Minimum-Weight, Mutually Exclusive Sets
Source: PLoS Comput Biol. 2015 Aug 28;11(8):e1004257. doi: 10.1371/journal.pcbi.1004257 (PMC4552843; doi:10.1371/journal.pcbi.1004257)

Figure S2 – A) Example of genes that are co-amplified with RNF139 and MYC in TCGA OV tumors. B) Expressions of RNF139 in TCGA OV tumors with or without RNF139 amplification. C) Expressions of MYC in TCGA OV tumors with or without MYC amplification.

A)

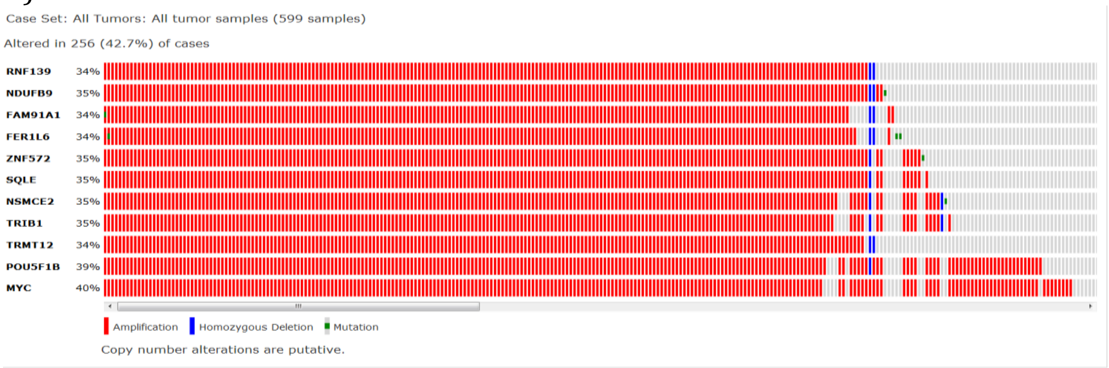

B)

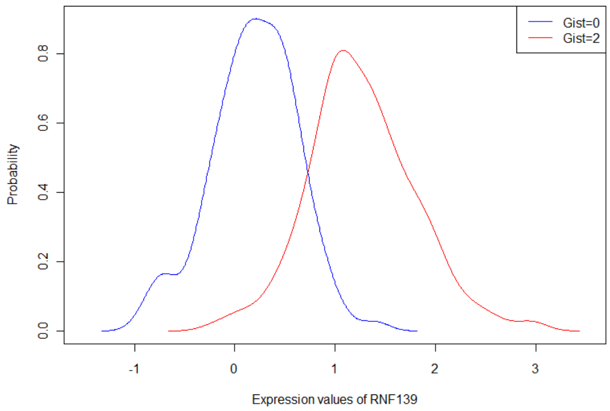

C)

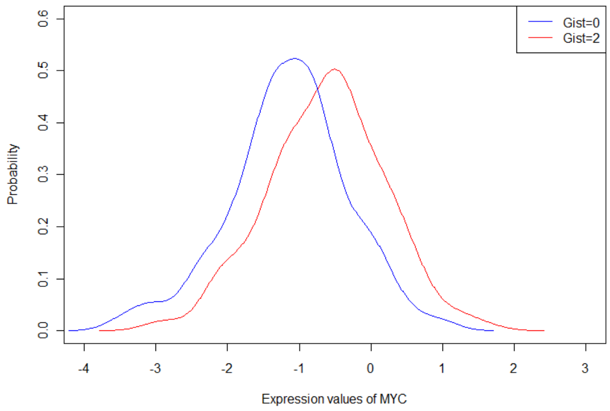

Supplement: S2 Fig — B) Expressions of RNF139 in TCGA OV tumors with or without RNF139 amplification. C) Expressions of MYC in TCGA OV tumors with or without MYC amplification. (PDF) [file pcbi.1004257.s009.pdf]
